# High-efficiency production of 5-hydroxyectoine using metabolically engineered *Corynebacterium glutamicum*

**DOI:** 10.1186/s12934-022-02003-z

**Published:** 2022-12-28

**Authors:** Lukas Jungmann, Sarah Lisa Hoffmann, Caroline Lang, Raphaela De Agazio, Judith Becker, Michael Kohlstedt, Christoph Wittmann

**Affiliations:** Institute of Systems Biotechnology, Saarland University, Campus A1.5, Saarbrücken, Germany

**Keywords:** *Corynebacterium glutamicum*, Extremolyte, Ectoine, 5-Hydroxyectoine, Ectoine hydroxylase, Biotransformation, Intracellular metabolite, Proline, Trehalose, High-value product

## Abstract

**Background:**

Extremolytes enable microbes to withstand even the most extreme conditions in nature. Due to their unique protective properties, the small organic molecules, more and more, become high-value active ingredients for the cosmetics and the pharmaceutical industries. While ectoine, the industrial extremolyte flagship, has been successfully commercialized before, an economically viable route to its highly interesting derivative 5-hydroxyectoine (hydroxyectoine) is not existing.

**Results:**

Here**,** we demonstrate high-level hydroxyectoine production, using metabolically engineered strains of *C. glutamicum* that express a codon-optimized, heterologous *ectD* gene, encoding for ectoine hydroxylase, to convert supplemented ectoine in the presence of sucrose as growth substrate into the desired derivative. Fourteen out of sixteen codon-optimized *ectD* variants from phylogenetically diverse bacterial and archaeal donors enabled hydroxyectoine production, showing the strategy to work almost regardless of the origin of the gene. The genes from *Pseudomonas stutzeri* (PST) and *Mycobacterium smegmatis* (MSM) worked best and enabled hydroxyectoine production up to 97% yield. Metabolic analyses revealed high enrichment of the ectoines inside the cells, which, *inter alia*, reduced the synthesis of other compatible solutes, including proline and trehalose. After further optimization, *C.* *glutamicum Ptuf ectD*^*PST*^ achieved a titre of 74 g L^−1^ hydroxyectoine at 70% selectivity within 12 h, using a simple batch process. In a two-step procedure, hydroxyectoine production from ectoine, previously synthesized fermentatively with *C. glutamicum ectABC*^*opt*^, was successfully achieved without intermediate purification.

**Conclusions:**

*C. glutamicum* is a well-known and industrially proven host, allowing the synthesis of commercial products with granted GRAS status, a great benefit for a safe production of hydroxyectoine as active ingredient for cosmetic and pharmaceutical applications. Because ectoine is already available at commercial scale, its use as precursor appears straightforward. In the future, two-step processes might provide hydroxyectoine de novo from sugar.

**Supplementary Information:**

The online version contains supplementary material available at 10.1186/s12934-022-02003-z.

## Background

Extremophilic organisms accumulate and secrete extremolytes, small organic metabolites that protect them at high salinity, high temperature, and in other extreme habitats [1–4]. The two most prominent extremolytes are non-proteinogenic cyclic amino acids, namely ectoine (1,4,5,6-tetrahydro-2-methyl-4-pyrimidinecarboxylic acid) and its hydroxylated derivative 5-hydroxyectoine (1,4,5,6-tetrahydro-2-methyl-5-hydroxy-4-pyrimidinecarboxylic acid, named hydroxyectoine here) [1, 5]. Both molecules provide excellent cosmotropic [1], protein- and DNA-stabilizing [4, 6, 7], as well as cell- and tissue-protecting and moisturizing properties [4, 6–8], which easily explains why they are of increasing commercial interest. Notably, the two ectoines are applied in high-price sectors such as cosmetics [9, 10], medicine [11–13] and biotechnology [14–17]. Ectoine is the industrial flagship among the world’s extremolytes. The compound is produced in commercial scale by the company bitop AG (Dortmund, Germany) using the natural producer *Halomonas elongata* under high-salinity conditions [4, 10, 18]. The product trades at approximately 1000 USD kg^−1^. In a rapidly growing market, ectoine has found its way into a range of products for human and animal health, including lung inhalation fluids, eye drops, nose sprays, and skin care products, among others.

An economically viable route to commercial hydroxyectoine is not existing to date, resulting in enormous prices around 17,000 USD kg^−1^ [5, 19]. The difficulty to produce hydroxyectoine, simply, is the unfavorable layout of the underlying biosynthetic pathway. Inevitably, hydroxyectoine is only accessible through the l-lysine pathway via ectoine as a central precursor [19] which results in the accumulation of mixtures of the two compounds, typically with larger shares of ectoine [20]. Although promising attempts demonstrated de novo hydroxyectoine synthesis, titers have remained low: 0.4 g L^−1^ in recombinant *C. glutamicum* [14], 2.9 g L^−1^ in the natural producer *Halomonas salina* [21], and 14.9 g L^−1^ in a massively engineered recombinant *Escherichia* *coli* strain that required a quite complex fermentation process [22]. The low titers make fermentation and downstream processing too costly. An alternative approach, previously suggested to derive hydroxyectoine, is based on bioconversion from ectoine through hydroxylation [23]. Heterologous expression of ectoine hydroxylase in *E. coli* enabled the conversion of an admittedly low amount of 2.1 g L^−1^ ectoine into hydroxyectoine within 24 h. Clearly, hydroxyectoine production has not advanced beyond an initial proof-of-principle stage.

**Fig. 1:**
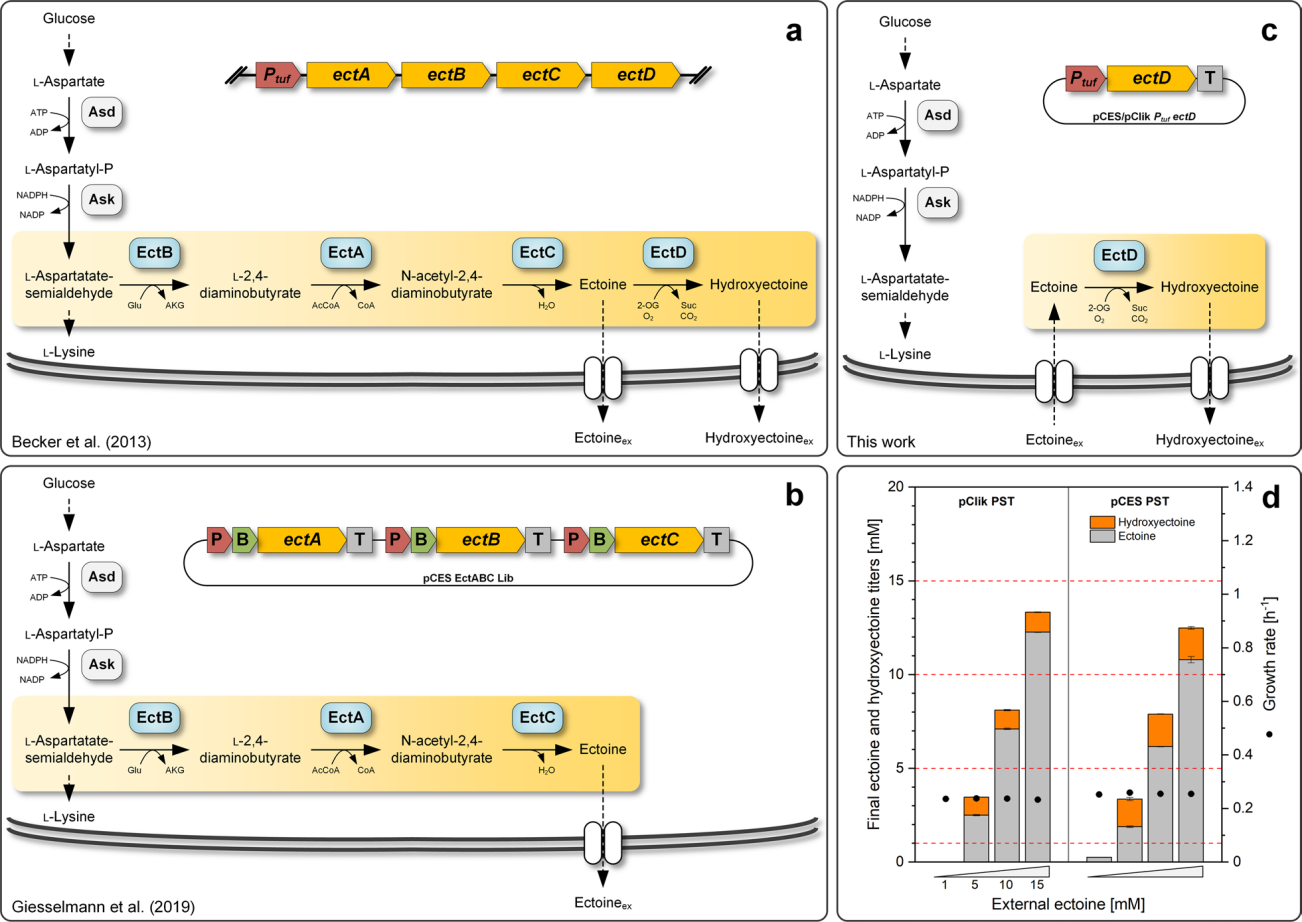
Genetic and metabolic engineering strategies to produce ectoine and hydroxyectoine in *C. glutamicum*. As part of the l-lysine biosynthetic pathway, l-aspartate is converted into l-aspartate-semialdehyde, involving aspartokinase (Ask; EC: 2.7.2.4) and l-aspartyl phosphate dehydrogenase (Asd; EC: 1.2.1.11). Synthesis of ectoines branches off from the l-aspartate-semialdehyde pool. First, l-2,4-diaminobutyrate transaminase (EctB; EC: 2.6.1.76) catalyzes the transamination to l-2,4-diaminobutyrate. Then, l-2,4-diaminobutyrate acetyltransferase (EctA; EC: 2.3.1.178) acetylates the intermediate to N-acetyl-2,4-diaminobutyrate. Finally, ectoine is cyclized by ectoine synthase (EctC; EC: 4.2.1.108). A fourth enzyme, i. e. ectoine hydroxylase (EctD; EC: 1.14.11), further converts ectoine into 5-hydroxyectoine via O_2_-dependent hydroxylation, simultaneously decarboxylating the co-substrate α-ketoglutarate. Previously, over-production of a mixture of ectoine and 5-hydroxyectoine in engineered *C. glutamicum* ECT-2 [14] was achieved by genomic integration of the (codon-optimized) *ectABCD* cluster from *Pseudomonas stutzeri* under control of the promoter *P*_*tuf*_ (**a**). Pure ectoine production in *C. glutamicum ectABC*^*opt*^ [15] was based on episomal monocistronic expression of the codon-optimized *ect*^*ABC*^ genes from *P. stutzeri*, each under control of a distinct promoter (P), a bicistronic element (B) and a terminator sequence (T), respectively (**b**). Metabolic engineering strategy of this work to convert ectoine to hydroxyectoine in a biotransformation set-up using recombinant *C. glutamicum* that expresses *ectD* under control of *P*_*tuf*_ (**c**). Experimental proof of hydroxyectoine production using the biotransformation set-up. The *C. glutamicum* type strain ATCC 13032 episomally expressed the codon optimized *ectD* gene from *Pseudomonas stutzeri* A1501 (PST) in the pClik 5a (pClik) and pCES-PLPV (pCES) vector. Cultures were grown at 30 °C on minimal glucose medium in a microbioreactor with different initial levels of supplied ectoine (1, 5, 10, 15 mM) and analyzed for growth (on-line measurement of OD_620_) and the conversion of ectoine into 5-hydroxyectoine (final titers after depletion of glucose) (**d**). n = 2

Previously, our group has metabolically engineered the soil microbe *C. glutamicum* to overproduce different l-lysine-based chemicals, including l-lysine itself [24], diaminopentane [25], glutarate [26], 5-aminovalerate [27], and, last but not least, mixtures of ectoine and hydroxyectoine [14] as well as pure ectoine [15]. The latter two studies provided valuable proof-of-concept that *C. glutamicum* expresses heterologous proteins that convert l-aspartate semialdehyde, an intermediate of l-lysine biosynthesis [28], into the two ectoines (Fig. 1a, b).

Here, we describe novel *C.* *glutamicum* cell factories that express ectoine hydroxylase to transform ectoine into its derivative hydroxyectoine. After screening for the most powerful enzyme and several rounds of strain and process optimization, up to 75 g L^−1^ hydroxyectoine was finally obtained in a batch process within only 12 h, providing an important next step towards upgrading this precious molecule to the industrial level.

## Results

### *C. glutamicum* converts externally supplied ectoine into hydroxyectoine upon heterologous expression of the *ectD* gene from *P. stutzeri*

As previously observed, *C. glutamicum* ECT-2 co-produced ectoine and hydroxyectoine upon expression of a genomic copy of the *ectABCD* operon from *P. stutzeri* (PST) [14]. This finding provided an important pre-requisite for the envisioned biotransformation in the microbe: functional operation of the desired ectoine hydroxylase.

In a first experiment we examined if *C. glutamicum*, expressing *ectD* from *P. stutzeri*, can convert externally supplied ectoine into hydroxyectoine and, beneficially, excrete the latter (Fig. 1c). For this purpose, the wild type was transformed with two different episomal plasmids, pClik *P*_*tuf* _*ectD*^*PST*^ and pCES *P*_*tuf* _*ectD*^*PST*^, differing in the plasmid backbone but each expressing the same codon-optimized *ectD* under control of *P*_*tuf*_, the native promoter of the *tuf* gene, enabling strong constitutive expression [29, 30]. The two mutants were grown in a miniaturized and parallelized reactor system. After 24 h of incubation at 30 °C in minimal glucose medium, supplemented with 1 mM ectoine, hydroxyectoine, however, was not detected, suggesting on a first glance that the bioconversion had failed (Fig. 1d). Surprisingly, ectoine was virtually not detected too. The solute was apparently taken up. Because the cells had no capacity to degrade ectoine, it therefore appeared possible that the desired ectoine hydroxylation took place inside the cells. However, even this had happened, the product was apparently not secreted, although the microbe was basically capable to export it [14].

When evaluated at higher initial ectoine level (5, 10, 15 mM), both strains, beneficially, secreted hydroxyectoine into the medium (Fig. 1d). Approximately 20—30% of the added ectoine was converted within 24 h. For initially 5 mM ectoine as example, up to 1.5 mM hydroxyectoine was secreted. Notably, in all cases, a substantial amount of the initially supplied ectoine could not be recovered. Both plasmids worked equally well. Cell fitness was not affected in any of the set-ups, as the specific growth rate remained constant throughout. Overall, the results provided an important proof of concept, but the conversion efficiency was rather low. The 30% hydroxyectoine content, achieved at best, left space for further optimization.

### Alternative ectoine hydroxylases from *M. smegmatis*, *H. elongata*, *and V. salexigens* enable hydroxyectoine production in *C. glutamicum*

**Fig. 2:**
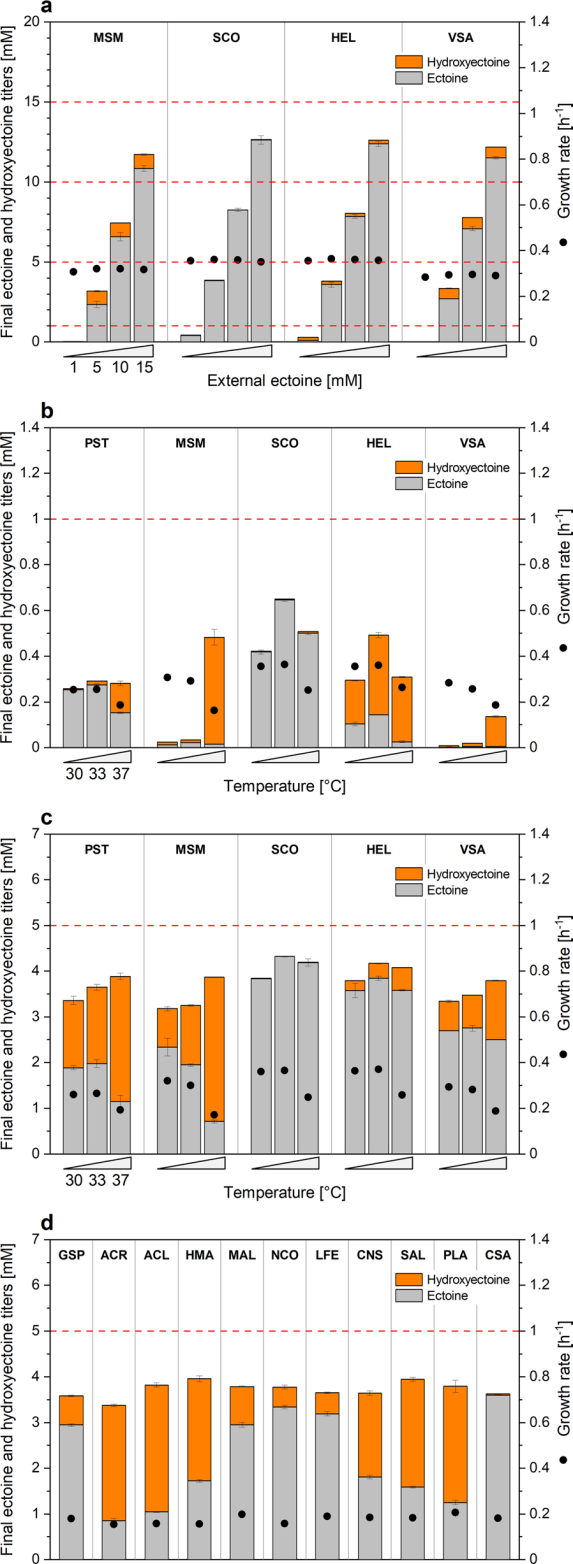
Screening for optimal 5-hydroxyectoine production in recombinant *C. glutamicum*. The *C. glutamicum* type strain ATCC 13032 episomally expressed the codon optimized *ectD* genes from *Pseudomonas stutzeri* A1501 (PST), *Mycobacterium smegmatis* ATCC 19420 (MSM), *Streptomyces coelicolor* A3(2) (SCO), *Halomonas elongata* ATCC 33173 (HEL) and *Virgibacillus salexigens* ATCC 700290 with amino acid exchanges A163C and S244C (VSA), in the pCES-PLPV vector. Cultures were grown at 30 °C on minimal glucose medium in a microbioreactor and analyzed for growth (on-line measurement of OD_620_) and the conversion of ectoine into hydroxyectoine (final titers after depletion of glucose). Screening at different initial ectoine concentrations (**a**), and different temperatures (**b**, **c**). n = 2. Screening of additional EctD enzyme variants in *C. glutamicum* at 37 °C and 5 mM ectoine (**d**). For the latter, *C.* *glutamicum* episomally expressed the codon optimized *ectD* genes from *Gracilibacillus sp.* SCU50 (GSP), *Acidiphilium cryptum* JF5 (ACR), *Alkalihalobacillus clausii* 7520–2 (ACL), *Hydrocarboniclastica marina* KCTC 62334 (HMA), *Methylomicrobium alcaliphilum* DSM 19304 (MAL), *Neptunomonas concharum* LHW37 (NCO), *Leptospirillum ferriphilum* ML-04 (LFE), *Candidatus Nitrosopumilus sp.* AR2 (CNS), *Sphingopyxis alaskensis* DSM 13593 (SAL), *Paenibacillus lautus* E7593-69 (PLA) and *Chromohalobacter salexigens* DSM 3043 (CSA), in the pCES-PLPV vector. The cultures were grown at 37 °C on minimal glucose medium in a microbioreactor and analyzed for growth (on-line measurement of OD_620_) and the conversion of 5 mM initial ectoine into hydroxyectoine (final titers after depletion of glucose). n = 3

To improve the conversion, we tested a diverse set of other EctD variants for their performance. As donors for EctD, we selected the halophilic microbes *Halomonas elongata* (HEL), the industrial ectoine producer [10], and *Virgibacillus salexigens* (VSA), naturally accumulating mixtures of ectoine and hydroxyectoine [31]. For the latter, we focused on a mutated EctD version that had shown superior kinetic properties in vitro [32]. Furthermore, two Gram-positive natural (hydroxy)ectoine-producers were chosen because of their taxonomically close relationship to *C. glutamicum*: *M.* *smegmatis* (MSM) [33] and *S.* *coelicolor* (SCO) [34] (Additional file 1: Fig. S1). Codon-optimization of the *ectD* genes, cloning into the two vectors under control of *P*_*tuf*_, strain construction, and strain evaluation at different ectoine levels was carried out as before. Beneficially, most EctD variants enabled hydroxyectoine production (Fig. 2a). The pCES MSM, VSA, and HEL variants accumulated up to 1.0 mM, 0.7 mM, and 0.2 mM of the product, respectively. As exception, strains that expressed the SCO enzyme variant did not yield any hydroxyectoine. The latter eventually related to general difficulties in expressing genes from *Streptomyces* in other hosts or to an incompatibility of the enzyme with the chosen culture. Again, batches with 1 mM of ectoine had mostly no solutes left in the cultivation broth. An exception was observed for the EctD variant from *H. elongata*, which resulted in small amounts of hydroxyectoine in the medium, and EctD from *S. coelicolor*, where minor shares of ectoine were observed. As before, cell fitness remained unchanged over the increasing ectoine concentration. Strains expressing *ectD* on the episomal pClik plasmid performed similar to the pCES-based strains (Additional file 1: Fig. S2a).

### Increased temperature boosts hydroxyectoine production up to 97% yield

The initial conversions were done at 30 °C, the optimum growth temperature of *C. glutamicum* [35]. Enzymes, however, have their optimal temperature mostly between 30 to 40 °C [36]. Notably, hydroxyectoine is preferably formed at higher temperature, and this effect was also observed in *C. glutamicum* before [14]. Therefore, additional cultivations were conducted at 33 °C and 37 °C. Higher temperature strongly increased hydroxyectoine production (Fig. 2b, c). The MSM variant showed the overall best performance. At 37 °C it produced hydroxyectoine with 97% and 82% selectivity out of 1 and 5 mM supplied ectoine, respectively. The PST variant performed similarly well in presence of 5 mM of ectoine. Both, the HEL and VSA variants showed high selectivity for 1 mM of ectoine but did not scale up accordingly at higher level. At 37 °C, slight hydroxyectoine formation was also observed for the SCO variant, demonstrating that also this enzyme was functionally expressed in *C. glutamicum*, although its activity was far below that of the other candidates. Generally, increased temperature resulted in impaired cell viability and a declining growth rate (Fig. 2b, c). As before, the final titer of both ectoines did not sum up to the initially supplied concentration. Up to 1.5 mM of the overall pool was no longer detectable in the supernatant. Again, the overall performance was comparable between pClik- and pCES-based producers (Additional file 1: Fig. S2b, c). All following experiments were conducted using pCES strains.

### Under optimum conditions, ten out of eleven further ectoine hydroxylases from phylogenetically diverse bacterial and archaeal donors are suitable for hydroxyectoine production

Beyond the five tested *ectD* candidates, ectoine hydroxylases had been identified in 272 out of 6,428 bacterial genomes, indicating a wide distribution within the bacterial kingdom [36]. We were interested, if recombinant hydroxyectoine production in *C. glutamicum* would be restricted to selected EctD variants or if the strategy would (beneficially) work more generally. Using the optimal setup (5 mM ectoine, 37 °C), we evaluated a broader range of *ectD* variants from different bacterial and archaeal families: *Bacillaceae* (*Gracilibacillus sp.* SCU50, GSP; *Alkalihalobacillus clausii* 7520–2, ACL), *Paenibacillaceae* (*Paenibacillus lautus* E7593-6, PLA), Acetobacteraceae (*Acidiphilium cryptum* JF5, ACR), *Alteromonadaceae* (*Hydrocarboniclastica marina* KCTC 62334, HMA), Methylococcaceae (*Methylomicrobium alcaliphilum* DSM 19304, MAL), *Oceanospirillaceae* (*Neptunomonas concharum* LHW37, NCO), *Nitrospiraceae* (*Leptospirillum ferriphilum* ML-04, LFE), *Sphingomonadaceae* (*Sphingopyxis alaskensis* DSM 13593, SAL), Halomonadaceae (*Chromohalobacter salexigens* DSM 3043, CSA), and the archaeal family *Nitrosopumilaceae* (*Candidatus nitrosopumilus sp.* AR2 (CNS). Surprisingly, ten out of the eleven enzyme variants led to hydroxyectoine production (Fig. 2d). Even the archaeal gene, being phylogenetically highly different, enabled high-efficiency hydroxyectoine production. Among all *ectD* variants tested, the genes from the gram-positive bacteria *A. clausii* 7520–2, *P. lautus* E7593-6, and *M. smegmatis*, and that of the gram-negative *A. cryptum* JF5 and *P. stutzeri* worked best. Considering also the first screening round with four out of five genes that successfully worked, the strategy appeared generally feasible, almost regardless of the origin of the gene.

### Efficient hydroxyectoine synthesis requires well-growing cells

**Fig. 3:**
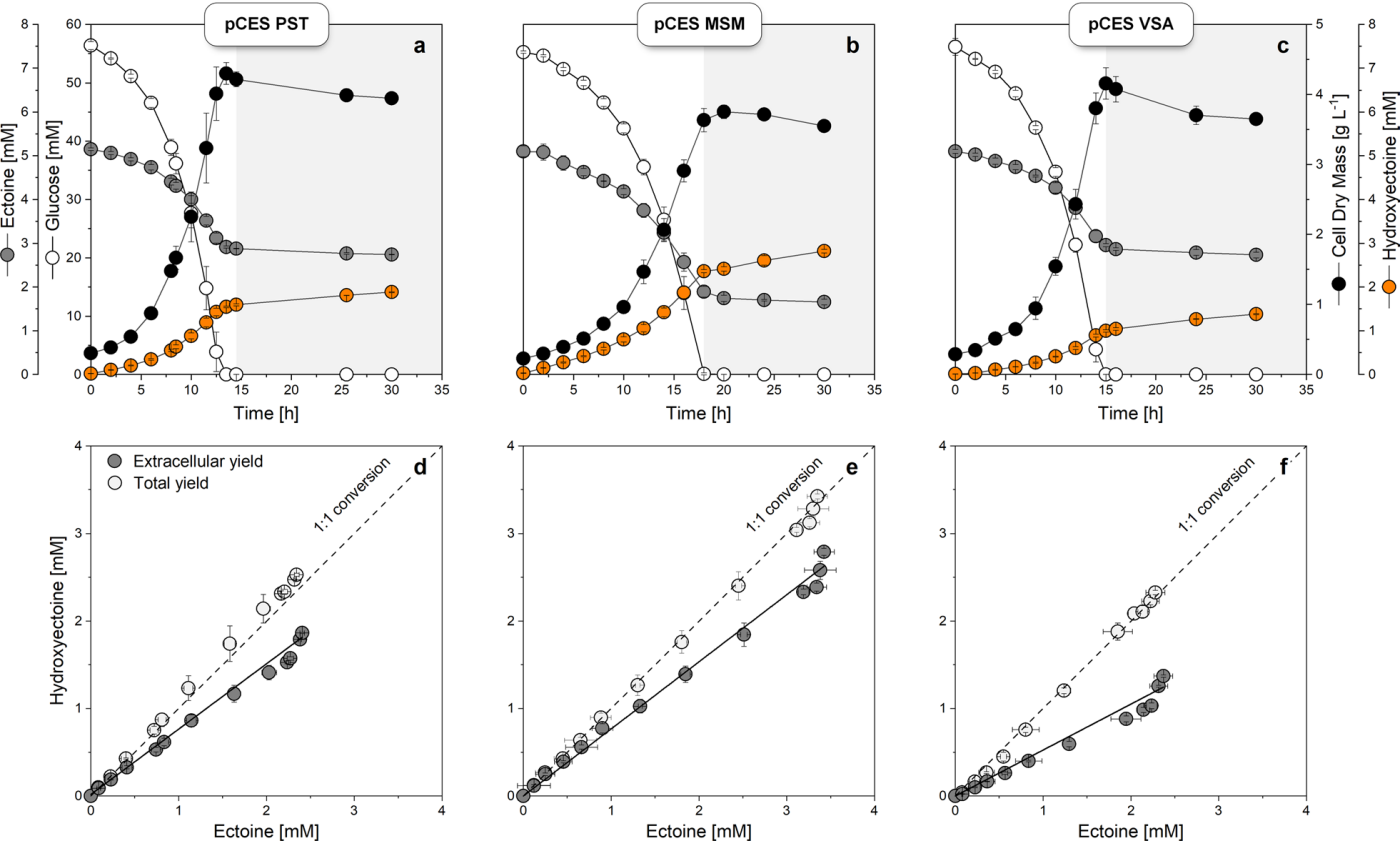
Kinetics and stoichiometry of 5-hydroxyectoine production in metabolically engineered strains of *C. glutamicum*. The *C. glutamicum* type strain ATCC 13032 episomally expressed the codon optimized *ectD* genes from *Pseudomonas stutzeri* A1501 (**a**, **c**), *Mycobacterium smegmatis* ATCC 19420 (**b**, **e**) and *Virgibacillus salexigens* ATCC 700290 with amino acid exchanges A163C and S244C (**c**, **f**) in the pCES-PLPV vector. All strains were cultivated in shake flasks on glucose minimal medium with 5 mM of ectoine at 37 °C. Dark grey circles show conversion based on extracellularly detectable ectoines. Light grey circles indicate conversion additionally considering intracellular levels of ectoines. n = 3

For further investigation, three strains *C. glutamicum P*_*tuf* _*ectD*^*MSM*^, *P*_*tuf* _*ectD*^*PST*^, *P*_*tuf* _*ectD*^*VSA*^ were monitored in shake flasks over time, using 37 °C with supplementation of 5 mM ectoine. Cells grew exponentially until glucose, the carbon and energy source, was fully consumed which happened after 15 to 17 h (Fig. 3). Ectoine conversion into hydroxyectoine started immediately and was growth-associated. After growth had stopped, cells continued to secrete hydroxyectoine at reduced level. However, ectoine was no longer consumed at this stage indicating that the increase was eventually due to an extra release from the cells. It was interesting to note that the three strains strongly differed in their production performance. The highest hydroxyectoine titer (2.8 mM) was achieved using the MSM-based EctD variant, 50% more than for the EctD from *P. stutzeri* and even 100% more than for the mutated EctD from *V. salexigens*. When inspecting the yield, i. e. produced hydroxyectoine versus consumed ectoine as estimated from supernatant analysis, (Fig. 3d-f), it turned out that none of the cell factories achieved the expected 1:1 ratio. In fact, the yield ranged from 0.51 over 0.68 to 0.74 mol mol^−1^ for the VSA, PST, and MSM strains, respectively. The correlation was linear, except for the final stage of the process, where an increase in the hydroxyectoine level was linked to a rather constant ectoine level.

### Ectoines are highly enriched inside the cells, whereby hydroxyectoine is preferred over ectoine

As a substantial fraction of the initially supplied ectoine was apparently missing at the end of the process while *C. glutamicum* was obviously not capable of degrading it, we evaluated if the solute (and also its derivative hydroxyectoine) remained inside the cells. In addition we searched for other metabolic changes, observed before e. g. in *Halomonas* sp. SBS 10 [37], *E. coli* [38], and *B. subtilis* [39] and providing valuable insights under genetic and environmental perturbation.

**Fig. 4:**
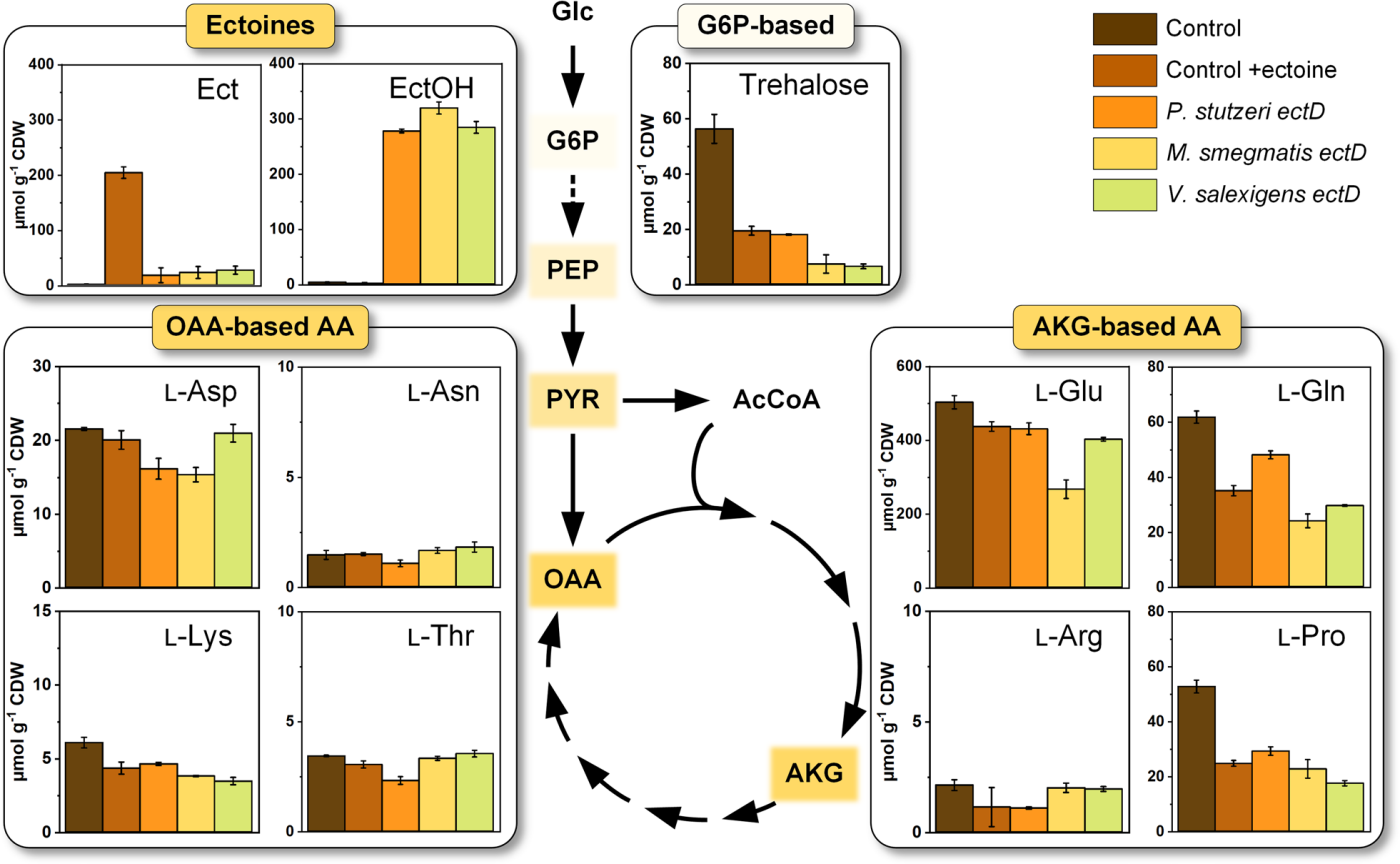
Impact of ectoine and 5-hydroxyectoine on intracellular metabolite levels in *C. glutamicum*. The *C. glutamicum* type strain ATCC 13032, episomally expressed the codon-optimized *ectD* genes from *Pseudomonas stutzeri* A1501, *Mycobacterium smegmatis* ATCC 19420 and *Virgibacillus salexigens* ATCC 700290 with amino acid exchanges A163C and S244C in the pCES-PLPV vector. All strains were cultivated in shake flasks on glucose minimal medium supplemented with 5 mM ectoine at 37 °C. Cells were harvested in mid-exponential phase (10 h) and disrupted for the analysis of intracellular metabolite levels. As a control, *C. glutamicum* harboring the empty vector pCES-PLPV was treated equally with (control + ectoine) and without (control) the addition of ectoine to the medium. n = 3

For this purpose, we extracted and quantified intracellular ectoine, hydroxyectoine, and free proteinogenic amino acids from the three strains during mid exponential growth, i. e. after 10 h (Fig. 3). *C. glutamicum*, carrying the empty pCES plasmid, served as reference. As expected, the glucose-grown control strain without ectoine addition did not contain intracellular ectoine and hydroxyectoine (Fig. 4). When cultivated on glucose in presence of 5 mM ectoine, however, *C. glutamicum* pCES exhibited a high intracellular ectoine level of around 200 µmol g_CDW_^−1^. Obviously, the strain efficiently took up the solute. The three ectoine hydroxylase-expressing strains pCES PST, MSM, and VSA, grown on glucose in the presence of ectoine, showed another, even more interesting picture. They all contained both, ectoine and hydroxyectoine. Remarkably, intracellular hydroxyectoine occurred at a huge level of 280 to 320 µmol g_CDW_^−1^. In comparison, ectoine was present at a much lower level of only 20 µmol g_CDW_^−1^ and thus accounted for only 5% of the total pool of the two ectoines. This finding was remarkable, because outside of the cells, ectoine was in excess at this stage of the culture (Fig. 3).

With regard to production, the intracellular pool displayed a substantial fraction of the totally synthetized hydroxyectoine. Considering this pool (and also the remaining intracellular ectoine) together with the extracellular levels provided the truly achieved yield of the conversion. When calculating this value, 100% conversion yield was obtained for all strains, i. e. each converted ectoine molecule yielded one hydroxyectoine molecule as expected from the pathway stoichiometry (Fig. 3d-f). It should be noted that, because of the strong intracellular accumulation, 25 to 50% of the product was kept inside the cells, suggesting breaking up the cells at the end of the process in order to collect all product formed.

### High intracellular levels of the ectoines coincide with decreased intracellular pools of amino acids of the α-ketoglutarate family

The accumulation of ectoine and hydroxyectoine inside the cells had consequences for the intracellular abundance of amino acids from the α-ketoglutarate family (Fig. 4). Already when adding ectoine to the non-producing control strain that carried the empty pCES vector, the pools of l-glutamic acid, l-glutamine, and l-proline were strongly reduced. The decline was also obvious for the three hydroxyectoine producers, with l-glutamic acid being reduced by up to 47% in the MSM strain. Likewise, intracellular levels of l-glutamine (up to 61%) and l-proline (up to 50%) dropped substantially. A similar pattern was found for l-histidine, where the concentrations were halved in the presence of solutes, while other amino acids did not change significantly in their abundance between strain and condition (Fig. 4, Additional file 1: Fig. S3).

### Benchmarking ***C. glutamicum P***_***tuf*** _***ectD***^***MSM***^ in a fed-batch process provides hydroxyectoine at high level from sucrose and ectoine

**Fig. 5:**
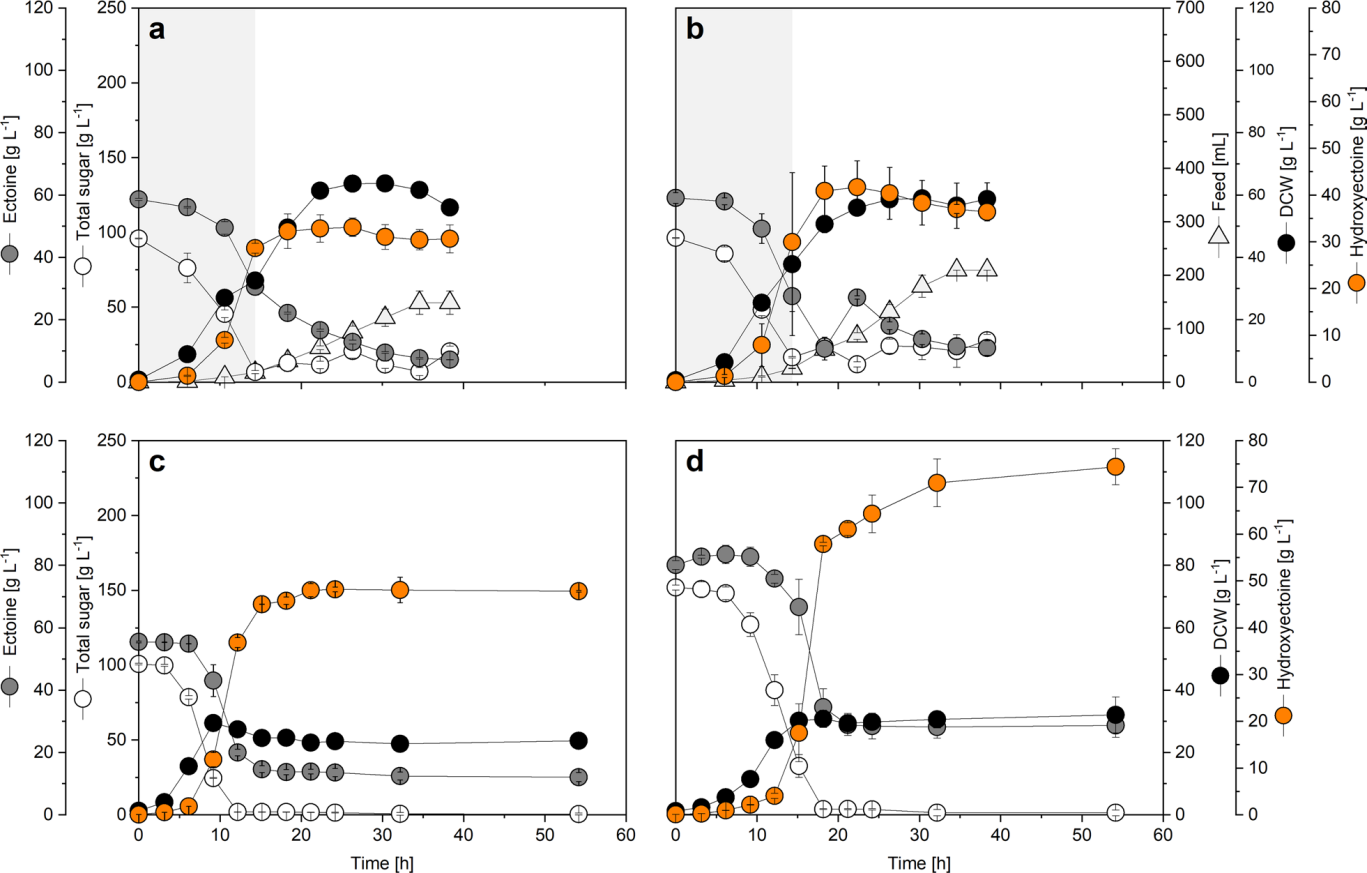
Production of 5-hydroxyectoine in stirred tank bioreactors using metabolically engineered *C. glutamicum*. The *C. glutamicum* type strain ATCC 13032 episomally expressed the codon optimized *ectD* genes from *Mycobacterium smegmatis* ATCC 19420 (**a**) and *Pseudomonas stutzeri* A1501 (**b**–**d**). Fed-batch-mode process at 37 °C with initial sucrose and ectoine concentrations of 100 g L^−1^ and 60 g L^−1^, and the addition of feed (600 g L^−1^ sucrose, 15 g L^−1^ yeast extract) at constant rate, respectively (**a**, **b**). Batch-mode process at 37 °C with initial sucrose and ectoine concentrations of 100 g L^−1^ and 50 g L^−1^ (**c**) and 150 g L^−1^ and 75 g L.^−1^, respectively (**d**). n = 2

The best performing strain in shake flask, *C.* *glutamicum P*_*tuf* _*ectD*^*MSM*^, was now evaluated in fed-batch mode (Fig. 5a). The process started from an initial level of 100 g L^−1^ of sucrose (as growth substrate), 60 g L^−1^ of ectoine (as precursor for the biotransformation), and 5 g L^−1^ yeast extract (to support cell vitality at 37 °C). The cells quickly consumed sucrose. The high sugar level used, obviously did not cause any inhibitory effect on cell growth. Hydroxyectoine was produced from early on and reached a level of 29 g L^−1^ after 15 h. Within this time, the batched sugar was almost completely consumed, and feeding was started at constant rate from a concentrated feed that contained 600 g L^−1^ sucrose plus a minor share of yeast extract (5 g L^−1^) which kept the sugar level in the bioreactor at about 10 g L^−1^, while the dissolved oxygen level was controlled at 30% saturation. During the feed phase, cell growth continued, and the biomass concentration reached a maximum of 60 g_CDW_ L^−1^ after 30 h. The hydroxyectoine level increased further during the initial feed period. A maximum hydroxyectoine titer of 33 g L^−1^ was reached after 26 h. Although the biotransformation continued further on, as visible from the still strong use of ectoine during this phase, the actual concentration of hydroxyectoine decreased during later stages, caused by the overlay of the weaker production with the dilution resulting from the added feed. However, the on-going conversion further enhanced the selectivity between ectoine and hydroxyectoine. Finally, hydroxyectoine was obtained at 79 mol-% selectivity.

In a series of further fermentations, we investigated other process conditions to eventually improve production performance (Additional file 1: Fig. S5). Two scenarios aimed at an increased supply of co-substrates required by the EctD protein, namely oxygen and l-glutamic acid [19]. Neither supplementation with l-glutamic acid during the batch or the feed phase nor the control of dissolved oxygen DO at a higher level of 60% was found beneficial. In fact, production remained rather unchanged. Likewise, the attempt to limit growth by omitting yeast extract and reducing nitrogen supply and, in this way, channel more cellular resources towards product formation, was not found successful. The retarded growth even negatively affected hydroxyectoine formation. A fourth set-up batched ectoine at only 10 g L^−1^ and then added periodic feed pulses to keep its concentration above 5 g L^−1^. It was, however, also less efficient and yielded only 19 g L^−1^ hydroxyectoine. In this way, we could successfully demonstrate conversion of ectoine into hydroxyectoine at higher scale. The standard fed-batch configuration provided the highest hydroxyectoine titer, reported to date.

### ***C. glutamicum P***_***tuf*** _***ectD***^***PST***^ accumulates 42 g L^−1^ hydroxyectoine in a fed-batch process

After bench-marking the *C. glutamicum P*_*tuf* _*ectD*^*MSM*^ strain, we wanted to also investigate the performance of the second-best strain *C. glutamicum P*_*tuf* _*ectD*^*PST*^ in fed-batch mode (Fig. 5b). The setup remained the same. As before, cells quickly consumed sucrose and feeding was started after 15 h to maintain the sugar level above 10 g L^−1^. At that time, 30 g L^−1^ of hydroxyectoine were present, comparable to the MSM strain. Likewise, biomass concentration rose up to 60 g_CDW_ L^−1^ after 30 h during the feed phase. However, hydroxyectoine production was comparably higher during the initial feed phase, with a titer of 41 g L^−1^ after 18 h. Due to the still on-going production with ectoine running short we added an additional ectoine shot after 19 h, to eventually maximize the outcome of the process. Over the next 20 h, the bioconversion of ectoine continued, however, with a constantly declining turnover rate. At last, selectivity of hydroxyectoine was 75 mol-%. The maximum hydroxyectoine titer of 42 g L^−1^ after 22 h, surpassed that of the MSM strain by 27%.

### In batch fermentation, driven by high substrate levels, ***C. glutamicum P***_***tuf*** _***ectD***^***PST***^ provides hydroxyectoine at a titer of 74 g L^−1^ and a selectivity of 70%

Albeit fed-batch fermentation led to the high hydroxyectoine titers, the feed part of the process added only slight benefits for the bioconversion. The batch phase, lasting only for one third of the total process time, provided already up to 87% of the maximum titer. Beneficially, the feed phase increased selectivity, but prolonged the process and later even diluted the product. Hence, we aimed to upgrade the process to be run faster and simpler. Inspired by the excellent tolerance of engineered *C. glutamicum* which efficiently used high sucrose levels, we shifted to a batch mode operation that was driven by high substrate levels. Three conditions that used different starting concentrations of sucrose and ectoine were evaluated: (i) 100 g L^−1^ and 50 g L^−1^ (100%), (ii) 150 g L^−1^ and 75 g L^−1^ (150%), and (iii) 200 g L^−1^ and 100 g L^−1^ (200%), respectively. To match the higher concentrations of sucrose and ectoine, all other components were increased to 150% and 200%, as compared to the first condition. In all scenarios, *C. glutamicum P*_*tuf *_*ectD*^*PST*^ was used (Fig. 5c, d, Additional file 1: Fig. S6).

The 100%-batch process started at 100 g L^−1^ sucrose and 50 g L^−1^ ectoine. Growth of strain *P*_*tuf *_*ectD*^*PST*^ set in immediately. The cells grew at a high rate of 0.41 h^−1^, which was even higher than that observed for the exponentially growing strain under moderate substrate levels (Fig. 2b, c). Accordingly, sucrose was consumed very fast. The sugar was depleted already after 12 h. At this time point, the hydroxyectoine titer had reached 37 g L^−1^, meaning that 60% of the ectoine had been converted. Remarkably, production continued after the cells had stopped growing and entered the stationary phase so that the hydroxyectoine titer increased to 48 g L^−1^ after 21 h which was 15% higher than the maximum value observed in the fed-batch before. The desired derivative was present at 78% selectivity (Table 2, Additional file 1: Fig. S7a).

Pleasingly, the 150%-batch process turned out to be even more efficient. Despite the higher sucrose level of 150 g L^−1^, cells started growth without any lag phase. The growth rate was slightly reduced to 0.25 h^−1^, so that the depletion of the sugar lasted somewhat longer than that in the lower concentrated batch, approximately 18 h. At this time point, the engineered cell factory had accumulated 58 g L^−1^ hydroxyectoine, and the product titer continued to rise even further later on. Finally, 74 g L^−1^ hydroxyectoine was obtained at a selectivity of 70% after 54 h (Table 2, Additional file 1: Fig. S7b). The most concentrated batch was less efficient (Additional file 1: Fig. S6e, f). Cell growth was rather low (0.08 h^−1^) so that the sucrose depletion took 54 h, far longer than for the other set-ups. Here, only 40 g L^−1^ hydroxyectoine was secreted at a lower selectivity of 33 mol-% (Table 2, Additional file 1: Fig. S7c). Obviously here, the substrate levels were so high that they reduced the vitality and metabolic activity of the cells.

### A two-step bioprocess with two complementary *C. glutamicum* cell factories allows *de-novo* production of hydroxyectoine from sugar

**Fig. 6:**
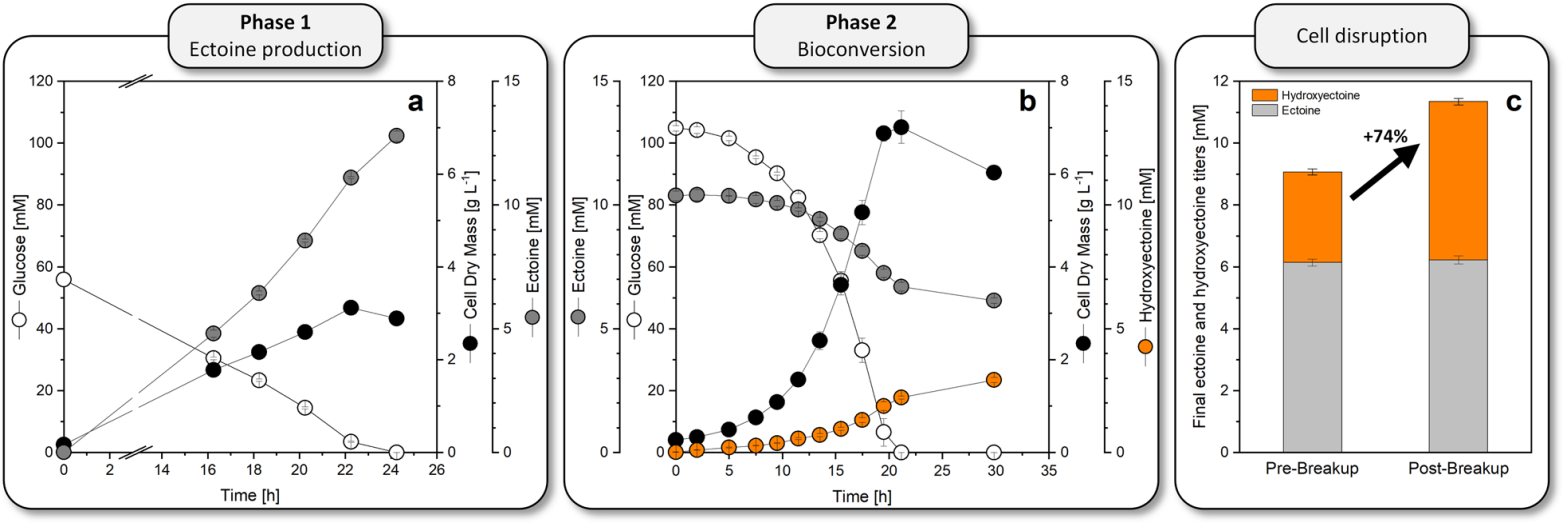
*De-novo* synthesis of hydroxyectoine using a two-step bioprocess. Step one: cultivation of the ectoine producer *C. glutamicum* *ectABC*^*opt*^ [15] on 10 g L^−1^ glucose minimal medium at 30 °C (**a**). Step two: cultivation of *C. glutamicum* episomally expressing the codon optimized *ectD* gene from *Mycobacterium smegmatis* ATCC 19420 at 37 °C. The medium displayed the final broth from step 1, clarified from the ectoine-producing cells, neutralized to pH 7.4, and replenished with glucose (20 g L.^−1^) (**b**). Final ectoine and hydroxyectoine titers of phase 2 before and after cell disruption (**c**). n = 3

Towards, a more cost effective and sustainable process, we aimed for hydroxyectoine production from sugar as the sole raw material, without supplementation of pre-purified ectoine. The chosen strategy involved a two-step fermentation setup, with de novo ectoine synthesis in the first step and ectoine bioconversion into hydroxyectoine in the second step, directly in the broth of the first fermentation. We applied the recently created cell factory *C. glutamicum ectABC*^*opt*^ [15] to produce ectoine in step one. Grown on minimal glucose medium (10 g L^−1^) at 30 °C, the strain accumulated 12.8 mM of ectoine within 24 h and, thereby, achieved a high yield of 0.23 mol mol^−1^ (Fig. 6a). Subsequently, the cells were removed. The obtained supernatant was neutralized and replenished with 20 g L^−1^ of glucose. Then, the medium was inoculated with *C. glutamicum P*_*tuf *_*ectD*^*MSM*^ and incubated further at 37 °C. Within 30 h, a significant fraction of hydroxyectoine was formed and secreted (Fig. 6b, Table 1).To recover intracellular product, cells were finally disrupted, increasing the final titer by 74% to 5.1 mM (Fig. 6c).

**Table 1:** Kinetics and stoichiometry of 5-hydroxyectoine of production from glucose and ectoine in *C.* *glutamicum P*_*tuf *_*ectD*^*PST*^, *P*_*tuf *_*ectD*^*MSM*^, and *P*_*tuf *_*ectD*^*VSA*^, referring to the data in Fig. 3

Strain	One-step biotransformation				Two-step de novo synthesis
	Control	PST	VSA	MSM	MSM
µ_max_ [h^−1^]	0.38 ± 0.05	0.23 ± 0.02	0.19 ± 0.02	0.17 ± 0.01	0.19 ± 0.00
Y_X/Glc_ [g mol^−1^]	62.20 ± 2.15	73.07 ± 3.06	69.73 ± 2.74	63.30 ± 1.90	68.23 ± 1.33
Y_EctOH/Ect_ [mol mol^−1^]	n.d.^a^	0.68 ± 0.01	0.51 ± 0.01	0.74 ± 0.03	0.56 ± 0.02
EctOH_max_ [mmol L^−1^]	n.d	1.88 ± 0.00	1.38 ± 0.01	2.82 ± 0.11	2.93 ± 0.10
Selectivity_EctOH_ [%]	n.d	40.72 ± 0.12	33.21 ± 0.29	62.36 ± 0.34	32.30 ± 1.06
Y_Tre/Glc_ [mmol mol^−1^]	4.97 ± 0.12	1.88 ± 0.00	6.13 ± 0.15	5.97 ± 0.15	6.40 ± 0.20

**Fig. 7:**
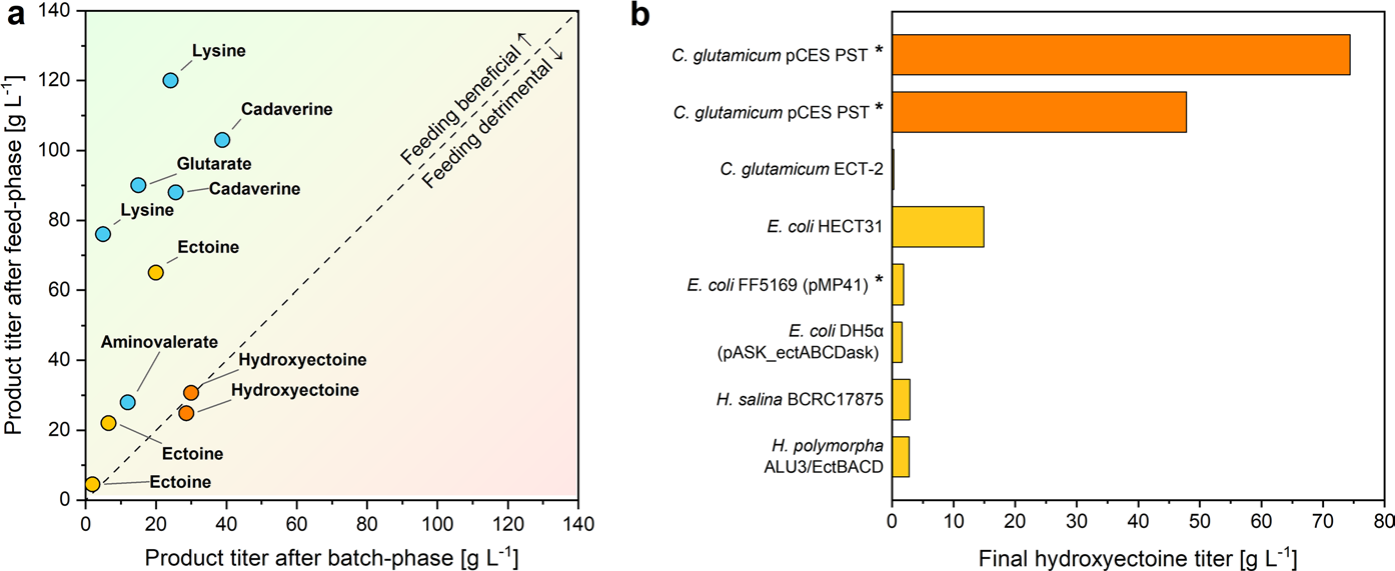
Benchmarking the newly developed 5-hydroxyectoine production process based on metabolically engineered *C.* *glutamicum.* Comparison of fed-batch processes using *C. glutamicum* to produce l-aspartate derived chemicals. Product titers, achieved after the initial batch-phase, are plotted against final titers, reached after the feed-phase. Blue circles: de novo production of l-lysine and products derived thereof [24–26, 30, 48, 86]; light orange circles: de novo synthesis of ectoine [14, 16]; dark orange circles: bioconversion from ectoine (this work) (**a**). Comparison of 5-hydroxyectoine titers of various microbial producers, namely *C. glutamicum* *P*_*tuf*_* ectD*^*PST*^ in a 1.5-fold (74.4 g L^−1^) and a onefold batch (47.8 g L^−1^) (this work), *C. glutamicum* ECT-2 (0.3 g L^−1^) [14], *E. coli* HECT31 (14.9 g L^−1^) [22], *E. coli* FF5169 pMP41 (1.9 g L^−1^) [23], *E. coli* DH5α pASK *ectABCDask* (1.6 g L^−1^) [87], *H. salina* BCRC17875 (2.9 g L^−1^) [21] and *H. polymorpha* ALU3/EctABCD (2.8 g L^−1^) [88]. Bioconversions are marked with stars

A non-producing control strain, containing the empty plasmid, is shown for comparison. Furthermore, the data in the right column show the performance of the second step of a two-step process (Fig. 7), based on fermented medium with de novo pre-synthetized ectoine, freshly supplemented with glucose. n = 3

^a^ Not detected

## Discussion

### ***C. glutamicum*** ***P***_***tuf*** _***ectD***^***PST***^ sets a milestone towards industrial hydroxyectoine production

Hydroxyectoine, the hydroxylated derivative of the industrial flagship ectoine seems to become the next star on the extremolyte market, given its unique properties [7, 40–42] and the proven economic value for these types of products, paved through ectoine [1]. As shown here, the expression of a codon-optimized copy of *ectD*, encoding ectoine hydroxylase, in the wild type of *C. glutamicum*, supported by bioprocess development, enabled high-level production of hydroxyectoine. *C. glutamicum* is a well-known, safe, and industrially proven host [27, 43–48]. The microbe produces no endotoxins, does not undergo phage lysis, and is generally recognized as safe (GRAS), allowing the synthesis of a range of commercial products granted GRAS status by the United States Food and Drug Administration for the food and pharmaceutical industries [44], which appears as a great benefit towards the safe, industrial-scale production of hydroxyectoine as active ingredient for cosmetic and pharmaceutical application.

During development, the mixed outcome from the evaluation of ectoine hydroxylases from several donors (Figs. 2, 3, Table 1) underlines the importance to screen different variants at the start [36], because only a few candidates matched with the cellular machinery of the host [23]. Hereby, the successful down-scaling of the process to microtiter plate scale at high reproducibility (Figs. 1d, 2, 3) appeared crucial to proceed fast and parallelized without being compromised in precision [49–51].

Using a simple batch process, the desired product was finally obtained at 74 g L^−1^, outcompeting all previous efforts, and exceeding the best titer reported so far, fivefold (Fig. 7). For this achievement, the product was transformed from ectoine, additionally adding sugar to support cell growth, a well-accepted process concept in chemical manufacturing [52]. From an industrial perspective, the demonstrated bioconversion strategy appears promising. First, it provides the product of interest at high efficiency. Second, because ectoine is already produced at the multi-ton level, it is available in sufficient amount to be converted into (currently not available) hydroxyectoine at large scale so that the novel product could be explored and commercialized further, including mode-of-action, and safety studies, formulation developments, as well as the preparation and evaluation of product samples [1, 53–55]. Production through bioconversion from ectoine, as demonstrated here, seems attractive to open the hydroxyectoine market. Similarly, bioconversion processes from simple precursors have helped to obtain other novel chemicals for facilitated market entry, prominent examples being the production of cadaverine [56] and 5-aminovalerate from l-lysine [57], as well as the production of γ-aminobutyric acid from l-glutamate [58].

### A two-stage fermentation process with two complementary *C. glutamicum* cell factories appears attractive to produce hydroxyectoine from sugar

Towards producing hydroxyectoine at even improved economic viability, there is no doubt that a simplified two-stage fermentation process without intermediate purification of ectoine would work as well and might finally display the strategy of choice. To this end, we built on our recently created strain *C. glutamicum ectABC*^*opt*^. This mutant, also based on *C. glutamicum* ATCC 13032, accumulated 65.2 g L^−1^ of ectoine *de-novo* from sugar [15] and thereby favorably approaches the level of 75 g L^−1^, found optimal for bioconversion in this work (Fig. 5d, Table 2). Here, we successfully demonstrated the 2-step bioconversion process is generally doable. With simple cell removal after the ectoine-producing stage, enrichment with fresh sugar, and inoculation with the novel bio-transformation cell factory *C. glutamicum* *P*_*tuf *_*ectD*^*MSM*^, developed here, hydroxyectoine was produced directly in the broth without need for transient ectoine purification. We believe that this two-step concept has huge potential for scale-up and further optimization to industrial scale. Because the two *C. glutamicum* strains are identical, except for the heterologous biosynthetic genes, they do not pose any extra demand on the used equipment so that e. g. the same seed line and the same production tank, even the same raw materials, could be used. Compared to direct *de-novo* biosynthesis of hydroxyectoine in one step [22], such a two-stage strategy would require approximately twice as much sugar. However, given the price of extremolytes of 1,000 USD per kg and more [5, 19], at least thousand-fold higher than the sugar price (1.1 USD per kg), extra substrate costs appear negligible here [59]. Moreover, at the same time, the double amount of sugar would deliver fivefold more hydroxyectoine (Fig. 7b). We would, however, like to state that we appreciate the metabolic engineering approach that demonstrated *de-novo* biosynthesis of hydroxyectoine in *E. coli* [22]. It is a nice showcase to selectively produce in a difficult pathway constellation. Although this strategy is less efficient at this stage, further strain engineering might help to improve performance (although this seems challenging as most things are already optimized out).

**Table 2:** Kinetics and stoichiometry of hydroxyectoine production from ectoine in *C.* *glutamicum P*_*tuf *_*ectD*^*PST*^ during batch fermentation with differently concentrated media

Strain	Onefold	1.5-fold	Twofold
µ_max_ [h^−1^]	0.41 ± 0.01	0.25 ± 0.00	0.08 ± 0.01
Y_X/Suc_ [g g^−1^]	0.32 ± 0.02	0.30 ± 0.00	0.13 ± 0.00
q_EctOH_ [g g^−1^ h^−1^]	0.12 ± 0.00	0.10 ± 0.01	0.04 ± 0.00
Y_EctOH/Ect_ [mol mol^−1^]	0.93 ± 0.03	0.99 ± 0.07	1.00 ± 0.04
EctOH_max_ [g L^−1^]	48.19 ± 0.52	74.42 ± 3.84	40.48 ± 4.00
Selectivity_EctOH_ [%]	78.08 ± 2.04	70.05 ± 1.75	33.15 ± 1.76

n = 3

As shown, metabolically engineered *C.* *glutamicum* exhibited highly selective production. At small scale, hydroxyectoine was produced at 97% selectivity, while the best-titer process still achieved 78% selectivity. This opens several opportunities. Notably, mixtures of hydroxyectoine and ectoine act as multifunctional anti-pollution agent [60]. Blends of the two molecules have been commercialized by the company bitop under the protected trade name 28Extremoin®. It appears straightforward to directly work up the obtained (roughly) 20:80 mixture of ectoine and hydroxyectoine, obtained here, towards such or similar blends. This would provide a high-value active ingredient at (i) high level, (ii) from a simple defined medium formulation, and (iii) without the need for complex and expensive separation of the two ectoines. The addition of (commercially available) ectoine would allow to adjust other extremolyte mixtures with likely different functional properties. Further optimization of strain and process might help to reach even higher hydroxyectoine levels towards a pure hydroxyectoine product.

### Efficient hydroxyectoine production requires actively growing cells

Many biotransformations can be conducted using non-growing cells [58, 61]. However, sufficient growth appeared crucial to drive efficient hydroxyectoine production in *C. glutamicum*. The conversion was efficient, as long as carbon was available, but quickly stopped, shortly after substrate depletion (Figs. 3, 5c, d), resulting in rather poor performance, when producing hydroxyectoine in a substrate-limited fed-batch process (Fig. 5a, b). Usually, the production of chemicals in *C.* *glutamicum* strongly benefits from fed-batch operation, where the feed-phase provides substantial amount of extra product (Fig. 7a), e. g. 80% in case of l-lysine [30] or even 225% in case of ectoine [15]. Moreover, hydroxyectoine producing strains were affected in growth (Table 1), which was previously not observed when expressing the *ectABC* operon using the same plasmid [15], excluding general expression-based defects but pointing to a specific metabolic burden that was caused by the novel enzyme. Notably, the hydroxylation, catalyzed by EctD, requires α-ketoglutarate and molecular oxygen as co-substrates [31]. Because oxygen was sufficiently available to the cells by efficient aeration, it seems that EctD (expressed to high level) competed for α-ketoglutarate with α-ketoglutarate dehydrogenase in the TCA cycle, the major catabolic pathway to generate energy and anabolic precursors [62]. The hydroxylation of ectoine consumed α-ketoglutarate so that carbon partially by-passed the TCA cycle, limiting the supply of energy and NADPH [19]. Thereby, the enzyme could have lowered the α-ketoglutarate pool, which, *inter alia*, would explain the lower pools of l-glutamate, l-glutamine, and related amino acids, all derived from this precursor (Fig. 5). More research seems interesting to resolve this picture further. Although growth might be affected too, future optimization could focus on a downregulation of α-ketoglutarate dehydrogenase, proven efficient to improve the production of l-glutamate [63] and putrescine [64].

### Hydroxyectoine is strongly retained in the cell

Extremolytes do not disturb but rather protect cellular processes, even at high level, whereby uptake is preferred over de novo synthesis [65, 66]. Here, *C. glutamicum* incorporated the externally supplied ectoine up to 200 µmol g_CDW_^−1^ (Fig. 4), comparable to *E. coli*, supplied with the solute at low salinity [67] and *C. glutamicum,* producing ectoine itself [14]. Astonishingly and observed for the first time, hydroxyectoine was accumulated to even higher level and displayed the major solute in the microbe. Obviously, *C. glutamicum* discriminated between the two ectoines. Eventually, the stronger tendency of hydroxyectoine to create hydrogen bonds might attach it stronger to cellular components [7, 68], or differences in import and export were involved.

Besides ectoines, amino acids and sugars are commonly used by microbes to counter osmotic stress. The most prominent ones are l-glutamate, l-glutamine, l-proline [69, 70], and trehalose, also in *C. glutamicum* [71]. Overall, microbes regulate their osmotic state with mixtures of several solutes that change in dependence of the environment [72–74]. This also includes the availability of external solutes and their uptake by the cell [66]. *C.* *glutamicum* was shown to reduce its l-proline pool when external betaine was available [75]. In line, all strains supplied with ectoine here, exhibited reduced levels of intracellular l-glutamate, l-glutamine, l-proline, and trehalose (Fig. 4). The reduced demand for the amino acids supported the production of hydroxyectoine due to the lower demand for a-ketoglutarate. Additionally, l-histidine was found reduced by 50% throughout all conditions (Additional file 1: Fig. S3). To date, this amino acid has not been considered as compatible solute to our knowledge, and we have no evidence that the molecule might really play such a role. Interestingly, its nitrogen-containing ring and its polar side chains, to some extent, resemble the structures of ectoine and l-proline, but more work is needed to substantiate this finding, remaining a bit peculiar at this stage.

## Conclusions

In this work, we demonstrate production of hydroxyectoine, a natural extremolyte with cell- and tissue protecting activity for high-value application as active ingredient in the cosmetic and pharmaceutical industries. As shown, recombinant *C. glutamicum* provided the desired product through heterologous expression of *ectD*, encoding for ectoine-hydroxylase. Using a simple batch process, the desired product was finally obtained at 74 g L^−1^, opening promising perspectives for commercial production of this precious molecule. In the future, as demonstrated in this work, two-stage processes that sequentially combine high-efficiency producers for ectoine from sugar [15] and hydroxyectoine from the pre-produced ectoine without purification plus fresh sugar might display a smart *de-novo* production scenario.

## Materials and methods

### Microorganisms and plasmids

*C. glutamicum* ATCC 13032 (DSM 20300) was obtained from the German Collection of Microorganisms and Cell Cultures (DSMZ, Braunschweig, Germany). For episomal expression, target genes were cloned into the shuttle vectors pClik 5α [76] and pCES-PLPV [77]. All strains and plasmids are listed in Table 3.

**Table 3:** *Corynebacterium glutamicum* strains and plasmids

Strains/Plasmids	Description	References
*Strains*		
E. coli DH10B	Heat shock-competent cells for vector amplification	Invitrogen
ATCC 13032 (DSM 20300)	Wild type of *C. glutamicum*	DSMZ
*C. glutamicum ectABC*^*opt*^	Ectoine producing strain	[15]
pCES	ATCC 13032 + pCES PLPV	This work
pCES PST	ATCC 13032 + pCES *P*_*tuf*_* ectD*_*PST*_	This work
pCES MSM	ATCC 13032 + pCES *P*_*tuf*_* ectD*_*MSM*_	This work
pCES SCO	ATCC 13032 + pCES *P*_*tuf*_* ectD*_*SCO*_	This work
pCES HEL	ATCC 13032 + pCES *P*_*tuf*_* ectD*_*HEL*_	This work
pCES VSA	ATCC 13032 + pCES *P*_*tuf*_* ectD*_*VSA*_	This work
pCES GSP	ATCC 13032 + pCES *P*_*tuf*_* ectD*_*GSP*_	This work
pCES ACR	ATCC 13032 + pCES *P*_*tuf*_* ectD*_*ACR*_	This work
pCES ACL	ATCC 13032 + pCES *P*_*tuf*_* ectD*_*ACL*_	This work
pCES HMA	ATCC 13032 + pCES *P*_*tuf*_* ectD*_*HMA*_	This work
pCES MAL	ATCC 13032 + pCES *P*_*tuf*_* ectD*_*MAL*_	This work
pCES NCO	ATCC 13032 + pCES *P*_*tuf*_* ectD*_*NCO*_	This work
pCES LFE	ATCC 13032 + pCES *P*_*tuf*_* ectD*_*LFE*_	This work
pCES CNS	ATCC 13032 + pCES *P*_*tuf*_* ectD*_*CNS*_	This work
pCES SAL	ATCC 13032 + pCES *P*_*tuf*_* ectD*_*SAL*_	This work
pCES PLA	ATCC 13032 + pCES *P*_*tuf*_* ectD*_*PLA*_	This work
pCES CSA	ATCC 13032 + pCES *P*_*tuf*_* ectD*_*CSA*_	This work
pClik PST	ATCC 13032 + pClik *P*_*tuf*_* ectD*_*PST*_	This work
pClik MSM	ATCC 13032 + pClik *P*_*tuf*_* ectD*_*MSM*_	This work
pClik SCO	ATCC 13032 + pClik *P*_*tuf*_* ectD*_*SCO*_	This work
pClik HEL	ATCC 13032 + pClik *P*_*tuf*_* ectD*_*HEL*_	This work
pClik VSA	ATCC 13032 + pClik *P*_*tuf*_* ectD*_*VSA*_	This work
*Plasmids*		
pCES PLPV	Episomal vector	[77]
pCES P_tuf_ ectD_PST_	Episomal vector with codon optimized *ectD* from *Pseudomonas stutzeri* A1501 under control of *Ptuf*	This work
pCES P_tuf_ ectD_MSM_	Episomal vector with codon optimized *ectD* from *Mycobacterium smegmatis* ATCC 19420 under control of *Ptuf*	This work
pCES P_tuf_ ectD_SCO_	Episomal vector with codon optimized *ectD* from *Streptomyces coelicolor* A3(2) under control of *Ptuf*	This work
pCES P_tuf_ ectD_HEL_	Episomal vector with codon optimized *ectD* from *Halomonas elongata* ATCC 33173 under control of *Ptuf*	This work
pCES P_tuf_ ectD_VSA_	Episomal vector with codon optimized *ectD* from *Virgibacillus salexigens* ATCC 700290 including amino acid exchanges A163C and S244C, under control of *Ptuf*	This work
pCES P_tuf_ ectD_GSP_	Episomal vector with codon optimized *ectD* from *Gracilibacillus sp.* SCU50 under control of *Ptuf*	This work
pCES P_tuf_ ectD_ACR_	Episomal vector with codon optimized *ectD* from *Acidiphilium cryptum* JF5 under control of *Ptuf*	This work
pCES P_tuf_ ectD_ACL_	Episomal vector with codon optimized *ectD* from *Alkalihalobacillus clausii* 7520–2 under control of *Ptuf*	This work
pCES P_tuf_ ectD_HMA_	Episomal vector with codon optimized *ectD* from *Hydrocarboniclastica marina* KCTC 62334 under control of *Ptuf*	This work
pCES P_tuf_ ectD_MAL_	Episomal vector with codon optimized *ectD* from *Methylomicrobium alcaliphilum* DSM 19304 under control of *Ptuf*	This work
pCES P_tuf_ ectD_NCO_	Episomal vector with codon optimized *ectD* from *Neptunomonas concharum* LHW37 under control of *Ptuf*	This work
pCES P_tuf_ ectD_LFE_	Episomal vector with codon optimized *ectD* from *Leptospirillum feriiphilum* ML-04 under control of *Ptuf*	This work
pCES P_tuf_ ectD_CNS_	Episomal vector with codon optimized *ectD* from *Candidatus Nitrosopumilus sp.* AR2 under control of *Ptuf*	This work
pCES P_tuf_ ectD_SAL_	Episomal vector with codon optimized *ectD* from *Sphyngopyxis alaskensis* DSM 13593 under control of *Ptuf*	This work
pCES P_tuf_ ectD_PLA_	Episomal vector with codon optimized *ectD* from *Paenibacillus lautus* E7593-69 under control of *Ptuf*	This work
pCES P_tuf_ ectD_CSA_	Episomal vector with codon optimized *ectD* from *Chromohalobacter salexigens* DSM 3043 under control of *Ptuf*	This work
pClik 5a MCS	Episomal vector	[76]
pClik P_tuf_ ectD_PST_	Episomal vector with codon optimized *ectD* from *Pseudomonas stutzeri* A1501 under control of *Ptuf*	This work
pClik P_tuf_ ectD_MSM_	Episomal vector with codon optimized *ectD* from *Mycobacterium smegmatis* ATCC 19420 under control of *Ptuf*	This work
pClik P_tuf_ ectD_SCO_	Episomal vector with codon optimized *ectD* from *Streptomyces coelicolor* A3(2) under control of *Ptuf*	This work
pClik P_tuf_ ectD_HEL_	Episomal vector with codon optimized *ectD* from *Halomonas elongata* ATCC 33173 under control of *Ptuf*	This work
pClik P_tuf_ ectD_VSA_	Episomal vector with codon optimized *ectD* from *Virgibacillus salexigens* ATCC 700290 including the amino acid exchanges A163C and S244C, under control of *Ptuf*	This work

### Molecular design and genetic engineering

To screen and evaluate a panel of ectoine hydroxylases, codon-optimized genes from *Mycobacterium smegmatis* ATCC 19420*, Virgibacillus salexigens* ATCC 700290 (comprising the amino acid exchanges A163C and S244C) [32]*, Streptomyces coelicolor* A3(2)*, Halomonas elongata* ATCC 33173, *Pseudomonas stutzeri* A1501, *Gracilibacillus sp.* SCU50, *Acidiphilium cryptum* JF5, *Alkalihalobacillus clausii* 7520–2, *Hydrocarboniclastica marina* KTC 62334, *Methylomicrobium alcaliphilum* DSM 19304, *Neptunomonas concharum* LHW37, *Leptospirillum ferriphilum* ML-04, *Candidatus Nitrosopumilus sp.* AR2, *Sphyngopyxis alaskensis* DSM 13593, *Paenibacillus lautus* E7593-69 and *Chromohalobacter salexigens* DSM 3043 were synthesized on basis of the digital sequence information (General Biosystems, Morrisville, NC, USA, Genscript Biotech Corp, Piscataway Township, NJ, USA). The obtained genes were amplified with specific primers (Additional file 1: Table S1) and cloned each under control of the constitutive *tuf* (NCgl0480) promoter [29] via Gibson Assembly [78] into the pClik 5α and pCES-PLPV vector, cut before with *Kpn*I/*Sal*I and *Sal*I/*Xba*I, respectively. Each gene was cloned into the pCES-PLPV vector, cut before with *Sal*I and *BamH*I, under control of the *tuf* promotor. The plasmids were then amplified in *E. coli* DH10B, isolated and transformed via electroporation into *C. glutamicum* [79]. Generally, the amplification of DNA fragments was done using PCR (2 × Phusion Flash PCR Master Mix, Thermo Fisher Scientific, Waltham, MA, USA, peQSTAR, PEQLAB Biotechnology GmbH, Erlangen, Germany). All created strains and plasmids were validated by PCR and Sanger sequencing (Genewiz, Leipzig, Germany). The sequences of the codon-optimized genes are given in Additional file 2.

### Growth media

Complex BHI medium (37 g L^−1^ brain heart infusion, BHI, Becton Dickinson, Franklin Lakes, NJ, USA) was used for first pre-cultures. The medium was amended with 20 g L^−1^ BD Difco agar (Becton Dickinson) for plate cultures. A minimal glucose medium was used for second pre-cultures and main cultures [80]. It contained per liter: 10 g of glucose, 1 g of NaCl, 0.055 g of Ca_2_Cl_2_·2H_2_O, 0.2 g of MgSO_4_·7H_2_O, 15 g of (NH_4_)_2_SO_4_, 24.98 g of K_2_HPO_4_, 7.7 g of KH_2_PO_4_, 20 mg of FeSO_4_·7H_2_O, 2 mg of FeCl_3_·6H_2_O, 2 mg of MnSO_4_·H_2_O, 1 mg of (NH_4_)_6_Mo_7_O_24_·4H_2_O, 0.5 mg of ZnSO_4_·H_2_O, 0.2 mg of CuCl_2_·2H_2_O, 0.2 mg of Na_2_B_4_O_7_·10H_2_O, 0.5 mg of biotin, 1 mg of thiamine·HCl, 1 mg of Ca-pantothenate, and 30 mg of 3,4-dihydroxybenzoic acid. For culturing strains with episomal plasmids, 50 mg L^−1^ of kanamycin was added from a filter-sterilized stock. Depending on the type of experiment, 99% ectoine (bitop, Dortmund, Germany) was added, as specified below. All ingredients were mixed freshly before use.

### Cultivation of *C. glutamicum* in shake flask

Cultures of *C. glutamicum* were incubated in baffled shake flasks (10% filling volume) in an orbital shaker at 230 rpm (Multitron, Infors AG, Bottmingen, Switzerland). For pre-culturing, cells were first grown overnight in complex BHI medium using a single colony from a 2-day pre-incubated BHI agar plate as inoculum. Subsequently, cells were harvested (8500 ×*g*, 5 min, RT), washed once with minimal medium, and used to inoculate a second pre-culture in minimal glucose medium. The second pre-culture was then harvested during the exponential phase (8500 ×*g*, 5 min, RT), washed once, and used to inoculate the main culture, again in minimal medium. The temperature was set to 30 °C for precultures and 37 °C for main cultures. All experiments were conducted as triplicate.

### Production of hydroxyectoine in a two-step bioconversion process in shake flask

In step one, *C. glutamicum ectABC*^*opt*^ [15] was cultivated in shake flasks as described above at 30 °C. After glucose depletion, cells were removed by centrifugation (10,000 x*g*, 10 min, RT). The pH of the obtained supernatant was adjusted to 7.4 (10 M NaOH) and the solution was once filtered (Filtropur S0.2, 0.2 µm, Sarstedt, Nümbrecht, Germany). Glucose was replenished to 20 g L^−1^ from a sterile 40% stock solution. In step two, *C. glutamicum P*_*tuf* _*ectD*^*MSM*^ was cultivated in the ectoine-containing medium from phase one in shake flasks as described above. To extract intracellular ectoine and hydroxyectoine, 1 mL culture sample was disrupted in Lysing Matrix B 2 mL tubes (MP Biomedicals, Solon, OH, USA) with a homogenisator (3 × 30 s, 6,500 rpm with 30 s break between cycles, Precellys® 24, Bertin Technologies, Montigny-le-Bretonneux, France) at. The supernatant was collected for HPLC analysis, conducted as described above. The process was conducted in triplicate.

### Cultivation of *C. glutamicum* in microbioreactors

Screening of *C. glutamicum* strains was carried out in 48-well flower plates with transparent bottom for online biomass sensing using a microbioreactor (BioLector, Beckman Coulter GmbH, Baesweiler, Germany, Baesweiler, Germany) at 1,300 rpm, 85% humidity and different temperatures, as given below. Two subsequent pre-cultures were conducted in shake flasks as described above. For main cultures, each well of the microtiter plate was filled with 1 mL of minimal glucose medium and inoculated to an initial OD_660_ of 0.5. Over the course of the cultivation, biomass was measured online as light backscatter at 620 nm. All experiments were conducted as triplicate.

### Fed-batch production of hydroxyectoine in stirred tank bioreactors

The best mutants were evaluated for their hydroxyectoine production capacity using fed-batch processes in 1 L bioreactors (SR0700DLS, DASGIP AG, Jülich, Germany). The initial batch medium (250 mL) contained per liter: 100 g of sucrose, 60 g of ectoine, 15 g of (NH_4_)_2_SO_4_ (pH 7.0), 5 g of yeast extract (Becton Dickinson), 2.07 g of citric acid, 1.25 g of KH_2_PO_4_, 1.25 g of Na_2_HPO_4_, 1.25 g of MgSO_4_·7H_2_O, 170 mg of CaSO_4_·2H_2_O, 30 mg of ZnSO_4_·7H_2_O, 14 mg MnSO_4_·H_2_O, 100 mg of FeSO_4_·7H_2_O, 30 mg of pantothenic acid, 9 mg of nicotinamide, 7.5 mg of thiamine·HCl, 4.5 mg of biotin, 0.4 mg of boric acid, 0.7 mg of CuSO_4_·5H_2_O, 0.6 mg of CoSO_4_·7H_2_O, 0.5 mg of NiSO_4_·6H_2_O, 0.06 mg of Na_2_MoO_2_·2H_2_O and 1 mL of antifoam 204 (Sigma-Aldrich). The process was inoculated from a pre-culture on BHI medium, supplemented with 50 µg ml^−1^ kanamycin, and incubated at 37 °C), and harvested during the mid-exponential growth phase by centrifugation (6,500 x*g*, 5 min, RT). The process was controlled at a temperature of 37 °C ± 0.1. The pH was monitored online using a pH electrode (Mettler Toledo 405-DPAS-SC-K8S/225, Mettler Toledo, Giessen, Germany) and maintained at 7.0 ± 0.1 by the automatic addition of 6 M NaOH and 6 M HCl (MP8 pump system, Eppendorf, Hamburg, Germany). To provide sufficient oxygen the pO_2_ level was measured online (Hamilton, Höchst, Germany) and maintained above a saturation of 30% by automatically adjusting stirrer speed and gas inflow. For data acquisition and process control the DASGIP control software was applied (DASGIP AG, Jülich, Germany).

The feed phase was initiated, when the initially added sucrose was consumed. Throughout the further process, manual feed pulses were added, when the dissolved oxygen level (pO_2_) surpassed 30% which indicated substrate depletion [26]. The feed solution contained per liter: 600 g of sucrose, 50 g of (NH_4_)_2_SO_4_ (pH 7.0), 5 g of yeast extract, 14 g of urea, 2.07 g of citric acid, 1.25 g of KH_2_PO_4_, 1.25 g of Na_2_HPO_4_, 1.25 g of MgSO_4_·7H_2_O, 170 mg of CaSO_4_·2H_2_O, 30 mg of ZnSO_4_·7H_2_O, 14 mg of MnSO_4_·H_2_O, 100 mg of FeSO_4_·7H_2_O, 30 mg of pantothenic acid, 9 mg of nicotinamide, 7.5 mg of thiamine·HCl, 4.5 mg of biotin, 0.4 mg of boric acid, 0.7 mg of CuSO_4_·5H_2_O, 0.6 mg of CoSO_4_·7H_2_O, 0.5 mg of NiSO_4_·6H_2_O, 0.06 of mg Na_2_MoO_2_·2H_2_O and 1 mL of antifoam 204 (Sigma-Aldrich). The fermentations were carried out in duplicate, except for processes that served for an evaluation of alternative conditions and were conducted as single replicate each.

### Batch production of hydroxyectoine in stirred tank bioreactors

Additionally, hydroxyectoine production was conducted in batch mode using 1 L bioreactors (SR0700DLS, DASGIP AG, Jülich, Germany). The preparation of the pre-cultures was handled as described above. Likewise, the other process settings were as described above. The batch process started with a volume of 300 mL. The standard batch medium contained per liter: 100 g sucrose, 50 g ectoine, 15 g (NH_4_)_2_SO_4_ (pH 7.0), 5 g yeast extract, 2.07 g citric acid, 1.25 g KH_2_PO_4_, 1.25 g Na_2_HPO_4_, 1.25 g MgSO_4_·7H_2_O, 170 mg CaSO_4_·2H_2_O, 30 mg ZnSO_4_·7H_2_O, 14 mg MnSO_4_·H_2_O, 100 mg FeSO_4_·7H_2_O, 30 mg pantothenic acid, 9 mg nicotinamide, 7.5 mg thiamine·HCl, 4.5 mg biotin, 0.4 mg boric acid, 0.7 mg CuSO_4_·5H_2_O, 0.6 mg CoSO_4_·7H_2_O, 0.5 mg NiSO_4_·6H_2_O, 0.06 mg Na_2_MoO_2_·2H_2_O and 1 mL antifoam. For testing the effect of higher sucrose and ectoine concentrations, higher concentrated batch media were used for comparison which contained 1.5-fold and twofold higher levels of all ingredients, respectively. All fermentations were carried out in duplicate.

### Quantification of intracellular metabolites

To quantify intracellular amino acids and ectoines, 2 mL of mid-exponentially growing cells were sampled by fast filtration (cellulose nitrate filter, 0.2 µm, 47 mm, Sartorius, Göttingen, Germany) [81], including twice washing of the cells on the filter with two volumes of 2.5% NaCl. The filters were then transferred into cups prefilled with a 220 µM α-amino butyric acid solution as internal standard for later quantification. For metabolite extraction, the suspended filters were boiled (100 °C, 15 min) and subsequently cooled on ice (2 min) before the extracts were transferred to reaction tubes and centrifuged (13,000 ×*g*, 5 min, 4 °C). The obtained supernatants were collected for analysis, and intracellular amino acids were quantified by HPLC [82].

### Quantification of ectoine and hydroxyectoine

Concentrations of ectoine and hydroxyectoine were analyzed by HPLC (Agilent Series 1290 Infinity, Agilent, Santa Clara, CA, USA). Separation was carried out at 20 °C on a RP-phase column (Inertsil ODS-3HP, 3.0 × 150 mm, 3.0 μm, GL Sciences BV, Eindhoven, The Netherlands) using 0.1% (v/v) perchloric acid as mobile phase at a flow rate of 0.5 mL min^−1^. The analytes were detected at a wavelength of 210 nm [83]. External standards were applied for quantification.

### Quantification of sugars and organic acids

Sugars (glucose, trehalose) and organic acids (lactate, acetate) were quantified using HPLC (Agilent Series 1260 Infinity, Agilent, Santa Clara, CA, USA) [24]. The analytes were separated at 40 °C by ion-moderated partitioning (Aminex HPX-87H, 300 × 7.8 mm, Bio-Rad, Hercules, CA, USA) using 5 mM H_2_SO_4_ as mobile phase at a flow rate of 0.5 mL min^−1^. Detection was carried out by refractive index measurement. External standards were used for quantification.

### Quantification of amino acids

Amino acid levels of cell lysates were quantified using HPLC (Agilent Series 1290 Infinity, Agilent, Santa Clara, CA, USA) with α-amino butyric acid as internal standard. For this, the analytes were derivatized with ortho-phthalaldehyde and 9-fluorenylmethyl chloroformate and were separated and quantified as described previously [84].

## Supplementary Information

**Additional file 1: Table S1.** Primers used for plasmid construction. **Figure S1.** Phylogenetic analysis of the donor organisms used in this work. The phylogenetic tree of the donor organisms and the host *C. glutamicum* ATCC 13032 was compiled using the phyloT online tool (biobyte solutions GmbH, Heidelberg, Germany) and trees visualized with the iTOL online tool [85]. **Figure S2.** Screening for optimal 5-hydroxyectoine production in recombinant *C. glutamicum*. The *C. glutamicum* type strain ATCC 13032 episomally expressed the codon optimized *ectD* genes from *Pseudomonas stutzeri* A1501 (PST), *Mycobacterium smegmatis* ATCC 19420 (MSM), *Streptomyces coelicolor* A3(2) (SCO), *Halomonas elongata* ATCC 33173 (HEL) and *Virgibacillus salexigens* ATCC 700290 with amino acid exchanges A163C and S244C (VSA) [32], in the pClik 5a (pClik) and pCES-PLPV (pCES) vector. Cultures were grown on minimal glucose medium in a microbioreactor and analyzed for growth (on-line measurement of OD_620_) and the conversion of ectoine into hydroxyectoine (final titers after depletion of glucose). Screening for different initial ectoine concentrations at 30 °C (a). Screening at different temperatures with 1 mM and 5 mM initial ectoine, respectively (b, c). n = 2. **Figure S3.** Impact of ectoine and 5-hydroxyectoine on intracellular metabolite levels in *C. glutamicum*. For 5-hydroxyectoine production *C. glutamicum* ATCC 13032, episomally expressed a codon-optimized *ectD* gene from *Pseudomonas stutzeri* A1501 (PST), *Mycobacterium smegmatis* ATCC 19420 (MSM), or *Virgibacillus salexigens* ATCC 700290 (VSA), respectively. The gene from *V. salexigens* encoded for an enzyme variant that carried the amino acid exchanges A163C and S244C [32]. All strains were cultivated at 37 °C on minimal glucose medium, supplemented with 5 mM ectoine. Cells were analyzed for intracellular metabolite levels during the mid-exponential phase (10 h). As a control, *C. glutamicum*, harboring the empty vector, investigated during growth on glucose and ectoine (Control) and during growth on glucose alone (Control -Ect), respectively. n = 3. **Figure S4.** Production of 5-hydroxyectoine in a fed-batch process in stirred tank bioreactors using metabolically engineered *C. glutamicum*. The *C. glutamicum* type strain ATCC 13032 episomally expressed the codon optimized *ectD* genes from *Mycobacterium smegmatis* ATCC 19420 (a, b) and *Pseudomonas stutzeri* A1501 (c, d). The process was operated in fed-batch-mode at 37 °C with initial sucrose and ectoine concentrations of 100 g L^−1^ and 60 g L^−1^, and the addition of feed (600 g L^−1^ sucrose, 15 g L^−1^ yeast extract) at constant rate, respectively. n = 2. **Figure S5.** Impact of process conditions on fed-batch production of 5-hydroxyectoine using strain *C. glutamicum* *P*_*tuf*_* ectD*^*MSM*^ in a stirred tank bioreactor at 37 °C. In each setup one process parameter was varied as compared to the standard layout (Fig. 7) to study its impact (from left to right): DO maintained at 60%, twice as high as in the control; supplementation of batch and feed medium with l-glutamate; limited nitrogen supply by elimination of small amounts of yeast extract from the batch medium and the use of a nitrogen-free feed; addition of ectoine at a reduced initial amount (10 g L^−1^) plus separate feeding later on. n = 1. **Figure S6.** Production of 5-hydroxyectoine in a batch process in stirred tank bioreactors using *C. glutamicum* *P*_*tuf*_* ectD*^*PST*^ which episomally expressed codon optimized *ectD* from *Pseudomonas stutzeri* A1501. The strain was cultivated in batch-mode with initial sucrose and ectoine concentrations of 100 g L^−1^ and 50 g L^−1^ (a, b), 150 g L^−1^ and 75 g L^−1^ (c, d) and 200 g L^−1^ and 100 g L^−1^ (e, f) at 37 °C. n = 2. **Figure S7.** Selectivity of 5-hydroxyectoine production in a batch process using *C. glutamicum* *P*_*tuf*_* ectD*^*PST*^. The *C. glutamicum* type strain ATCC 13032 episomally expressed codon-optimized *ectD* from *Pseudomonas stutzeri* A1501. It was cultivated with initial sucrose and ectoine concentrations of 100 g L^−1^ and 50 g L^−1^ (a), 150 g L^−1^ and 75 g L^−1^ (b), and 200 g L^−1^ and 100 g L^−1^ (c) at 37 °C. n = 2.

**Additional file 2: Table S1.** Native (original) and codon-optimized sequences of the used *ectD* genes.

## Data Availability

The dataset(s) supporting the conclusions of this article are all included within the article.

## Abbreviations

ACL: *Alkalihalobacillus clausii*
ACR: *Acidiphilium cryptum*
CDW: Cell dry weight
CNS: *Candidatus nitrosopumilus sp.*
CSA: *Chromohalobacter salexigens*
*ectD*: Gene encoding for ectoine hydroxylase
Ect: Ectoine
EctOH: 5-Hydroxyectoine
GSP: *Gracilibacillus sp.*
HEL: *Halomonas elongata*
HMA: *Hydrocarboniclastica marina*
LFE: *Leptospirillum ferriphilum*
MAL: *Methylomicrobium alcaliphilum*
MSM: *Mycobacterium smegmatis*
NCO: *Neptunomonas concharum*
PLA: *Paenibacillus lautus*
PST: *Pseudomonas stutzeri*
SAL: *Sphyngopyxis alaskensis*
SCO: *Streptomyces coelicolor*
*tuf*: Encoding gene of elongation factor TU
VSA: *Virgibacillus salexigens*
