# Supplementary material for: High-efficiency production of 5-hydroxyectoine using metabolically engineered Corynebacterium glutamicum
Source: Microb Cell Fact. 2022 Dec 28;21:274. doi: 10.1186/s12934-022-02003-z (PMC9798599; doi:10.1186/s12934-022-02003-z)
Supplement: Supplementary file 1 — Additional file 1: Table S1. Primers used for plasmid construction. Figure S1. Phylogenetic analysis of the donor organisms used in this work. The phylogenetic tree of the donor organisms and the host C. glutamicum ATCC 13032 was compiled using the phyloT online tool (biobyte solutions GmbH, Heidelberg, Germany) and trees visualized with the iTOL online tool [85]. Figure S2. Screening for optimal 5-hydroxyectoine production in recombinant C. glutamicum. The C. glutamicum type strain ATCC 13032 episomally expressed the codon optimized ectD genes from Pseudomonas stutzeri A1501 (PST), Mycobacterium smegmatis ATCC 19420 (MSM), Streptomyces coelicolor A3(2) (SCO), Halomonas elongata ATCC 33173 (HEL) and Virgibacillus salexigens ATCC 700290 with amino acid exchanges A163C and S244C (VSA) [32], in the pClik 5a (pClik) and pCES-PLPV (pCES) vector. Cultures were grown on minimal glucose medium in a microbioreactor and analyzed for growth (on-line measurement of OD620) and the conversion of ectoine into hydroxyectoine (final titers after depletion of glucose). Screening for different initial ectoine concentrations at 30 °C (a). Screening at different temperatures with 1 mM and 5 mM initial ectoine, respectively (b, c). n = 2. Figure S3. Impact of ectoine and 5-hydroxyectoine on intracellular metabolite levels in C. glutamicum. For 5-hydroxyectoine production C. glutamicum ATCC 13032, episomally expressed a codon-optimized ectD gene from Pseudomonas stutzeri A1501 (PST), Mycobacterium smegmatis ATCC 19420 (MSM), or Virgibacillus salexigens ATCC 700290 (VSA), respectively. The gene from V. salexigens encoded for an enzyme variant that carried the amino acid exchanges A163C and S244C [32]. All strains were cultivated at 37 °C on minimal glucose medium, supplemented with 5 mM ectoine. Cells were analyzed for intracellular metabolite levels during the mid-exponential phase (10 h). As a control, C. glutamicum, harboring the empty vector, investigated during growth on glucose and e [file 12934_2022_2003_MOESM1_ESM.docx]

**Additional file 1 to**

**High-efficiency production of 5-hydroxyectoine using**

**metabolically engineered Corynebacterium glutamicum**

*Microbial Cell Factories*

Lukas Jungmann, Sarah Lisa Hoffmann, Caroline Lang, Raphaela De Agazio, Judith Becker, Michael Kohlstedt, and Christoph Wittmann*

Institute of Systems Biotechnology, Campus A1.5, Saarland University, Saarbrücken, Germany

*Corresponding author: Phone: +49 681 302 71970, FAX: +49 681 302 71972, e‑mail: [christoph.wittmann@uni-saarland.de](mailto:christoph.wittmann@uni-saarland.de)

**Table S1:** Primers used for plasmid construction.

| **Plasmid** | **Primer** | **Sequence** |
| --- | --- | --- |
| pCES MSM | pCES MSM Ptuf fw | GGCCCCCCCTCGAGGTCGACTGGCCGTTACCCTGCGAATG |
|  | pCES MSM Ptuf rv | GTGAACTGGGTGGTGGTCATTGTATGTCCTCCTGGACTTC |
|  | pCES MSM ectD fw | GAAGTCCAGGAGGACATACAATGACCACCACCCAGTTCAC |
|  | pCES MSM ectD rv | TGGCCGGCTGGGCCTCTAGATTAAGTCACTGGGGCCACGC |
| pCES VSA | pCES VSA Ptuf fw | GGCCCCCCCTCGAGGTCGACTGGCCGTTACCCTGCGAATG |
|  | pCES VSA Ptuf rv | GATGGGTACAGGTCTTCCATTGTATGTCCTCCTGGACTTC |
|  | pCES VSA ectD fw | GAAGTCCAGGAGGACATACAATGGAAGACCTGTACCCATC |
|  | pCES VSA ectD rv | TGGCCGGCTGGGCCTCTAGATTAGTTCACTGCGGAGTACA |
| pCES SCO | pCES SCO Ptuf fw | GGCCCCCCCTCGAGGTCGACTGGCCGTTACCCTGCGAATG |
|  | pCES SCO Ptuf rv | TCCTGGCGTGGGGTTGCCACTGTATGTCCTCCTGGACTTC |
|  | pCES SCO ectD fw | GAAGTCCAGGAGGACATACAGTGGCAACCCCACGCCAGGA |
|  | pCES SCO ectD rv | TGGCCGGCTGGGCCTCTAGATTACTTCACTGGGGTGAAGT |
| pCES PST | pCES PST Ptuf fw | GGGAACAAAAGCTGGGTACCTGGCCGTTACCCTGCGAATG |
|  | pCES PST Ptuf rv | GGGTACAGATCTGCCTGCATTGTATGTCCTCCTGGACTTC |
|  | pCES PST ectD fw | GAAGTCCAGGAGGACATACAATGCAGGCAGATCTGTACCC |
|  | pCES PST ectD rv | GGCTGGGCCTCTAGAGTCGATTACAGGTACTGCTGTGGGC |
| pCES HEL | pCES HEL Ptuf fw | GGCCCCCCCTCGAGGTCGACTGGCCGTTACCCTGCGAATG |
|  | pCES HEL Ptuf rv | GAGGAGGTCTGCACGGACATTGTATGTCCTCCTGGACTTC |
|  | pCES HEL ectD fw | GAAGTCCAGGAGGACATACAATGTCCGTGCAGACCTCCTC |
|  | pCES HEL ectD rv | TGGCCGGCTGGGCCTCTAGATTAGCCGTCTGGGGACCAGG |
| pClik MSM | pClik MSM Ptuf fw | CTGACGTCGGGCCCGGTACCTGGCCGTTACCCTGCGAATG |
|  | pClik MSM Ptuf rv | GTGAACTGGGTGGTGGTCATTGTATGTCCTCCTGGACTTC |
|  | pClik MSM ectD fw | GAAGTCCAGGAGGACATACAATGACCACCACCCAGTTCAC |
|  | pClik MSM ectD rv | AGAAGAGCATCGATGTCGACTTAAGTCACTGGGGCCACGC |
| pClik VSA | pClik VSA Ptuf fw | CTGACGTCGGGCCCGGTACCTGGCCGTTACCCTGCGAATG |
|  | pClik VSA Ptuf rv | GATGGGTACAGGTCTTCCATTGTATGTCCTCCTGGACTTC |
|  | pClik VSA ectD fw | GAAGTCCAGGAGGACATACAATGGAAGACCTGTACCCATC |
|  | pClik VSA ectD rv | AGAAGAGCATCGATGTCGACTTAGTTCACTGCGGAGTACA |
| pClik SCO | pClik SCO Ptuf fw | CTGACGTCGGGCCCGGTACCTGGCCGTTACCCTGCGAATG |
|  | pClik SCO Ptuf rv | TCCTGGCGTGGGGTTGCCACTGTATGTCCTCCTGGACTTC |
|  | pClik SCO ectD fw | GAAGTCCAGGAGGACATACAGTGGCAACCCCACGCCAGGA |
|  | pClik SCO ectD rv | AGAAGAGCATCGATGTCGACTTACTTCACTGGGGTGAAGT |
| pClik PST | pClik PST Ptuf fw | CCTGACGTCGGGCCCGGTACTGGCCGTTACCCTGCGAATG |
|  | pClik PST Ptuf rv | GGGTACAGATCTGCCTGCATTGTATGTCCTCCTGGACTTC |
|  | pClik PST ectD fw | GAAGTCCAGGAGGACATACAATGCAGGCAGATCTGTACCC |
|  | pClik PST ectD rv | CAGAAGAGCATCGATGTCGATTACAGGTACTGCTGTGGGC |
| pClik HEL | pClik HEL Ptuf fw | CTGACGTCGGGCCCGGTACCTGGCCGTTACCCTGCGAATG |
|  | pClik HEL Ptuf rv | GAGGAGGTCTGCACGGACATTGTATGTCCTCCTGGACTTC |
|  | pClik HEL ectD fw | GAAGTCCAGGAGGACATACAATGTCCGTGCAGACCTCCTC |
|  | pClik HEL ectD rv | AGAAGAGCATCGATGTCGACTTAGCCGTCTGGGGACCAGG |


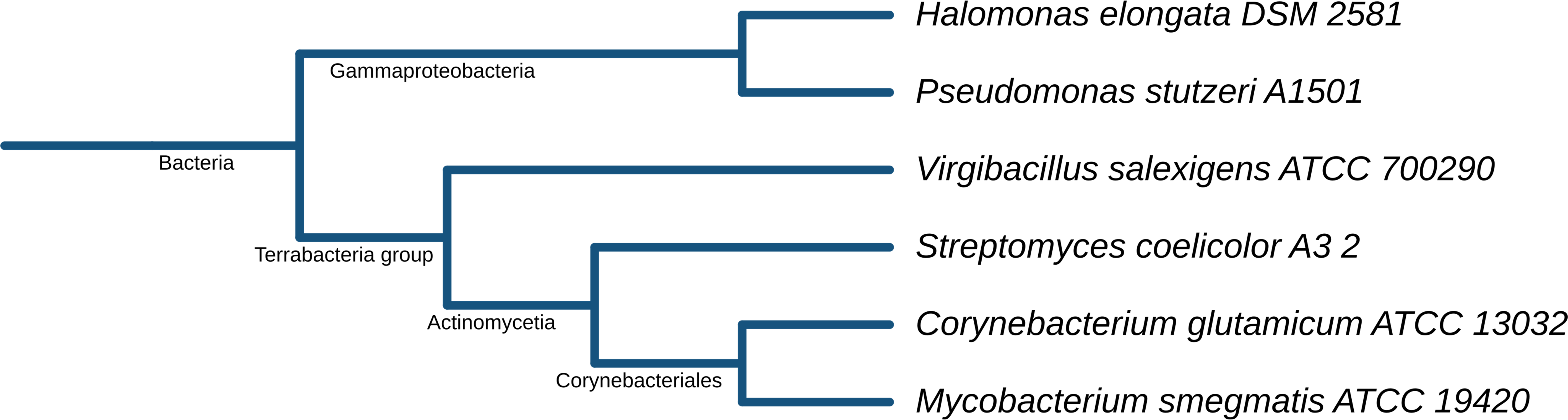


**Figure S1: Phylogenetic analysis of the donor organisms used in this work**. The phylogenetic tree of the donor organisms and the host *C. glutamicum* ATCC 13032 was compiled using the phyloT online tool (biobyte solutions GmbH, Heidelberg, Germany) and trees visualized with the iTOL online tool [[1](#_ENREF_1)].


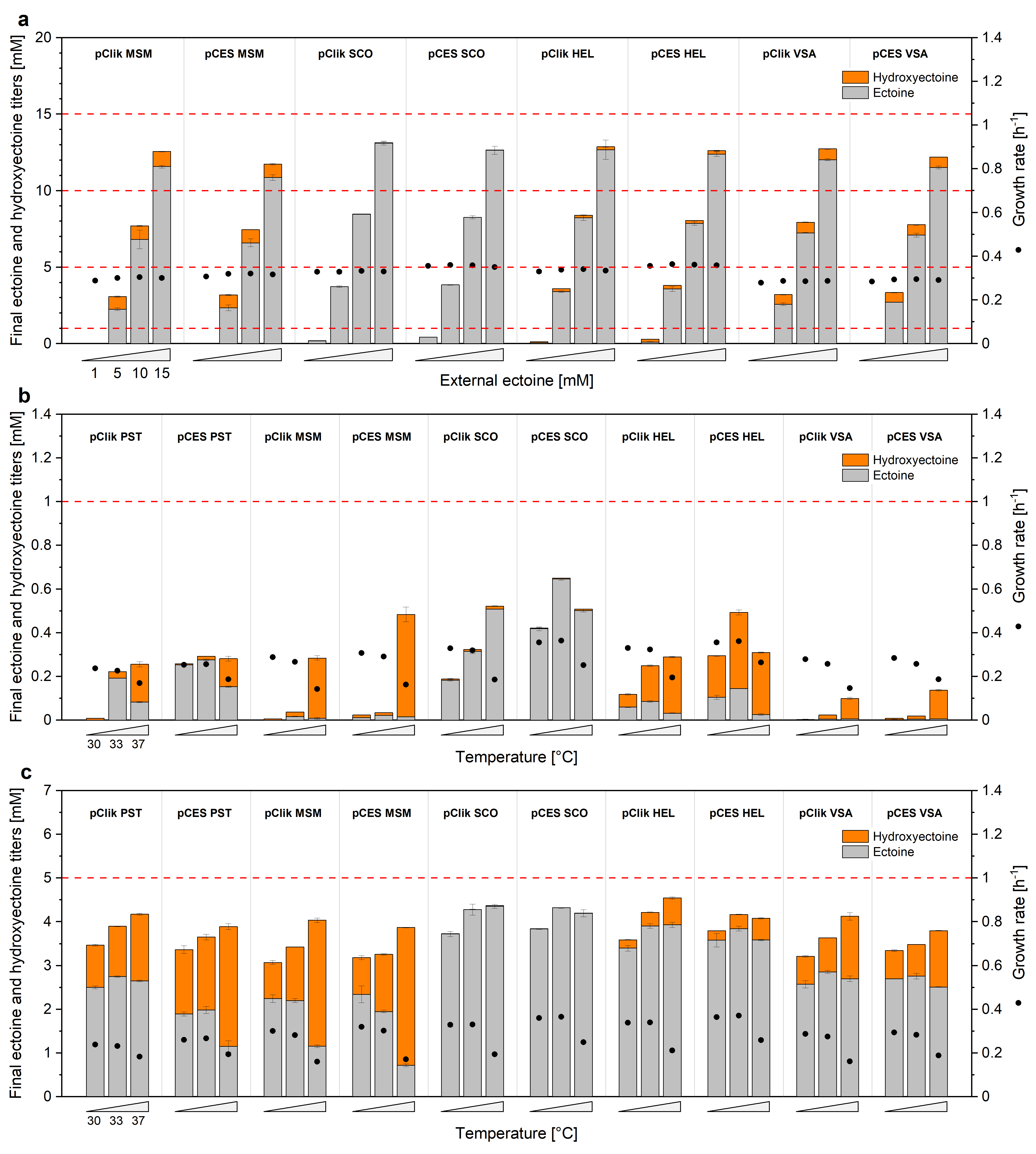


**Figure S2: Screening for optimal 5-hydroxyectoine production in recombinant *C. glutamicum*.** The *C. glutamicum* type strain ATCC 13032 episomally expressed the codon optimized *ectD* genes from *Pseudomonas stutzeri* A1501 (PST), *Mycobacterium smegmatis* ATCC 19420 (MSM), *Streptomyces coelicolor* A3(2) (SCO), *Halomonas elongata* ATCC 33173 (HEL) and *Virgibacillus salexigens* ATCC 700290 with amino acid exchanges A163C and S244C (VSA) [[2](#_ENREF_2)], in the pClik 5a (pClik) and pCES-PLPV (pCES) vector. Cultures were grown on minimal glucose medium in a microbioreactor and analyzed for growth (on-line measurement of OD_620_) and the conversion of ectoine into hydroxyectoine (final titers after depletion of glucose). Screening for different initial ectoine concentrations at 30°C (a). Screening at different temperatures with 1 mM and 5 mM initial ectoine, respectively (b, c). n=2.


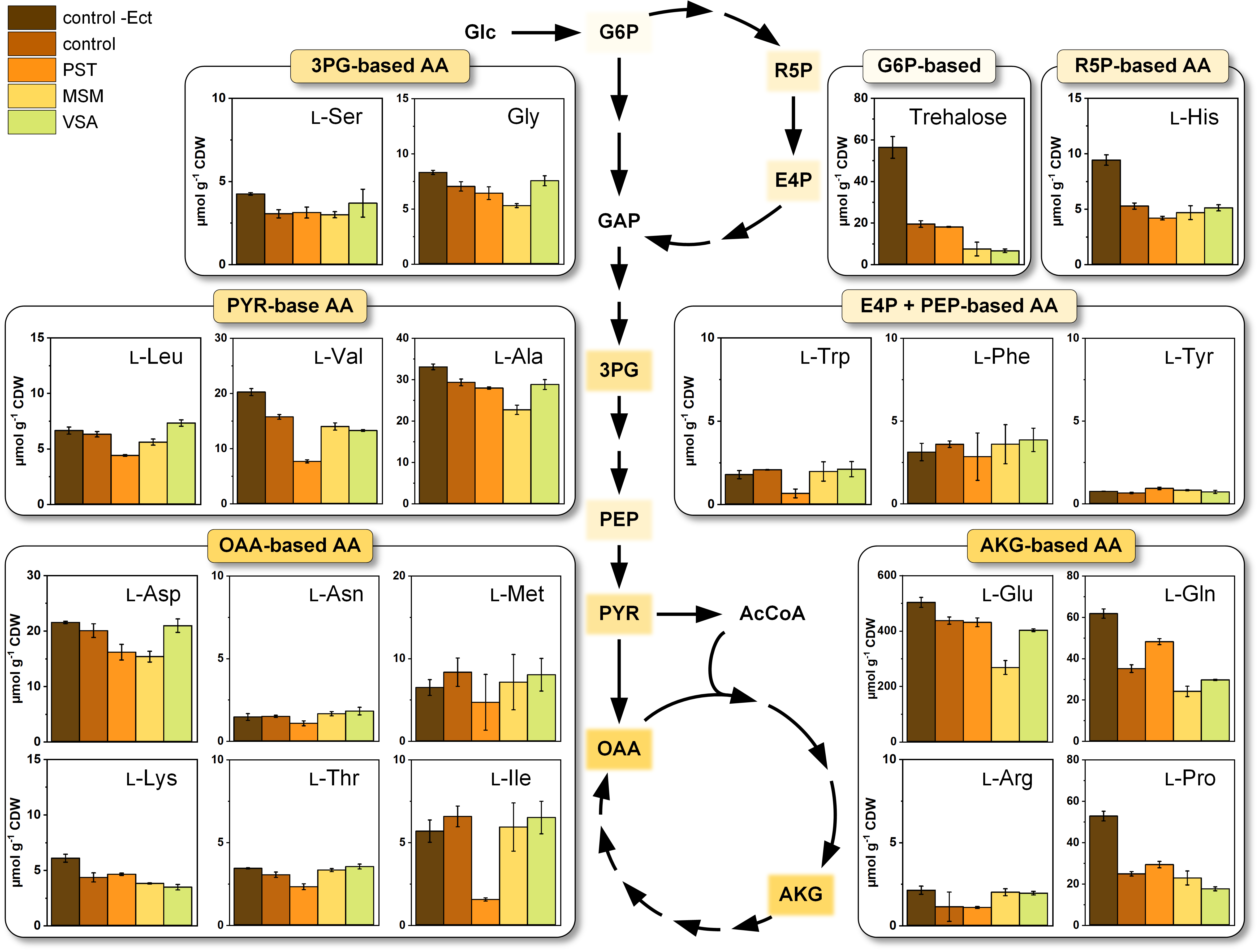


**Figure S3: Impact of ectoine and 5-hydroxyectoine on intracellular metabolite levels in *C. glutamicum*.** For 5-hydroxyectoine production *C. glutamicum* ATCC 13032, episomally expressed a codon-optimized *ectD* gene from *Pseudomonas stutzeri* A1501 (PST), *Mycobacterium smegmatis* ATCC 19420 (MSM), or *Virgibacillus salexigens* ATCC 700290 (VSA), respectively. The gene from *V. salexigens* encoded for an enzyme variant that carried the amino acid exchanges A163C and S244C [[2](#_ENREF_2)]. All strains were cultivated at 37°C on minimal glucose medium, supplemented with 5 mM ectoine. Cells were analyzed for intracellular metabolite levels during the mid-exponential phase (10 h). As a control, *C. glutamicum*, harboring the empty vector, investigated during growth on glucose and ectoine (Control) and during growth on glucose alone (Control -Ect), respectively. n=3

**
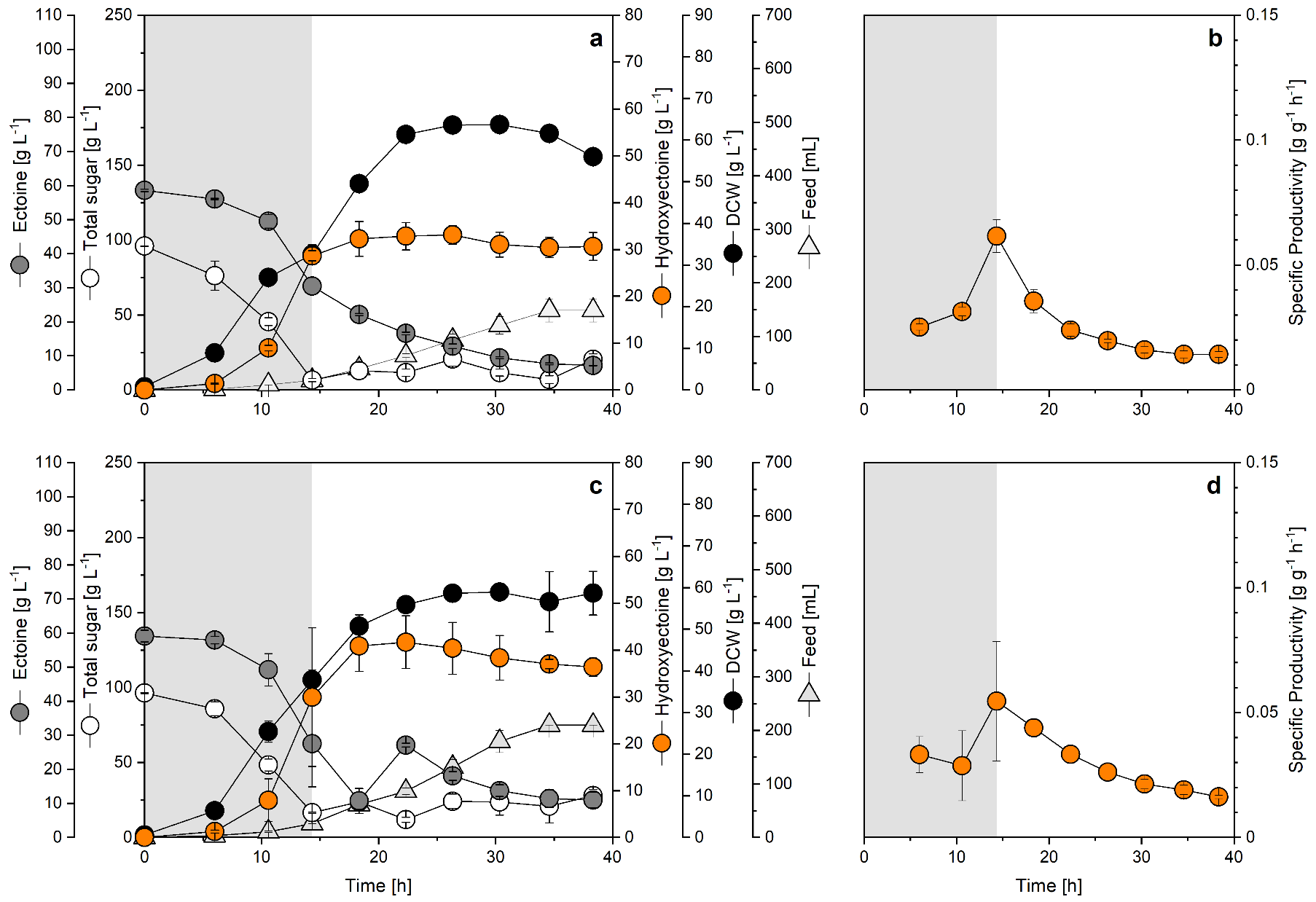
**

**Figure S4: Production of 5-hydroxyectoine in a fed-batch process in stirred tank bioreactors using metabolically engineered *C. glutamicum*.** The *C. glutamicum* type strain ATCC 13032 episomally expressed the codon optimized *ectD* genes from *Mycobacterium smegmatis* ATCC 19420 (a, b) and *Pseudomonas stutzeri* A1501 (c, d). The process was operated in fed-batch-mode at 37°C with initial sucrose and ectoine concentrations of 100 g L^-1^ and 60 g L^-1^, and the addition of feed (600 g L^-1^ sucrose, 15 g L^-1­^ yeast extract) at constant rate, respectively. n=2


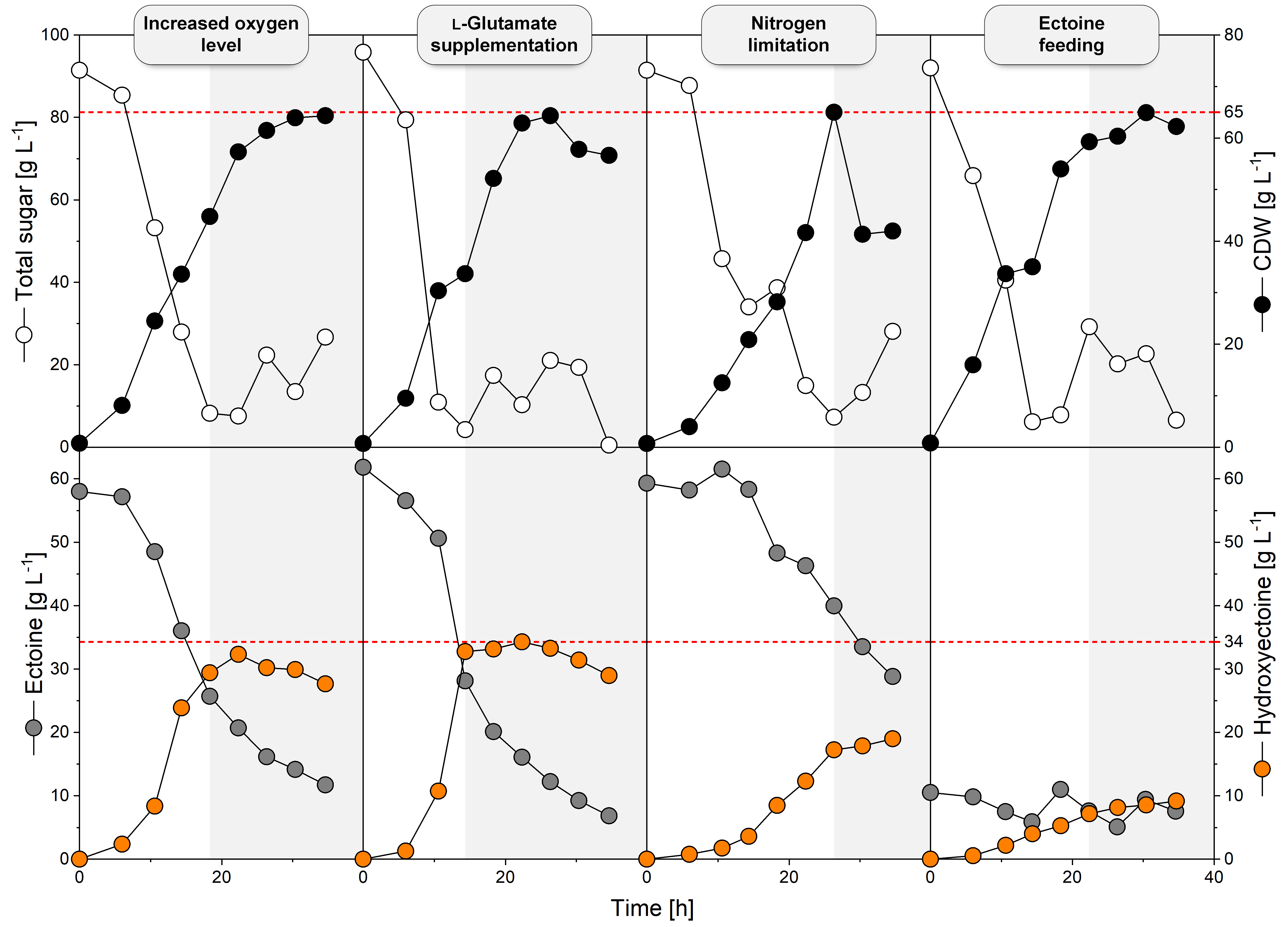


**Figure S5:** **Impact of process conditions on fed-batch production of 5-hydroxyectoine using strain *C. glutamicum* *P_tuf_* *ectD^MSM^* in a stirred tank bioreactor at 37°C.**In each setup one process parameter was varied as compared to the standard layout (Fig. 7) to study its impact (from left to right): DO maintained at 60%, twice as high as in the control; supplementation of batch and feed medium with l-glutamate; limited nitrogen supply by elimination of small amounts of yeast extract from the batch medium and the use of a nitrogen-free feed; addition of ectoine at a reduced initial amount (10 g L^-1^) plus separate feeding later on. n=1


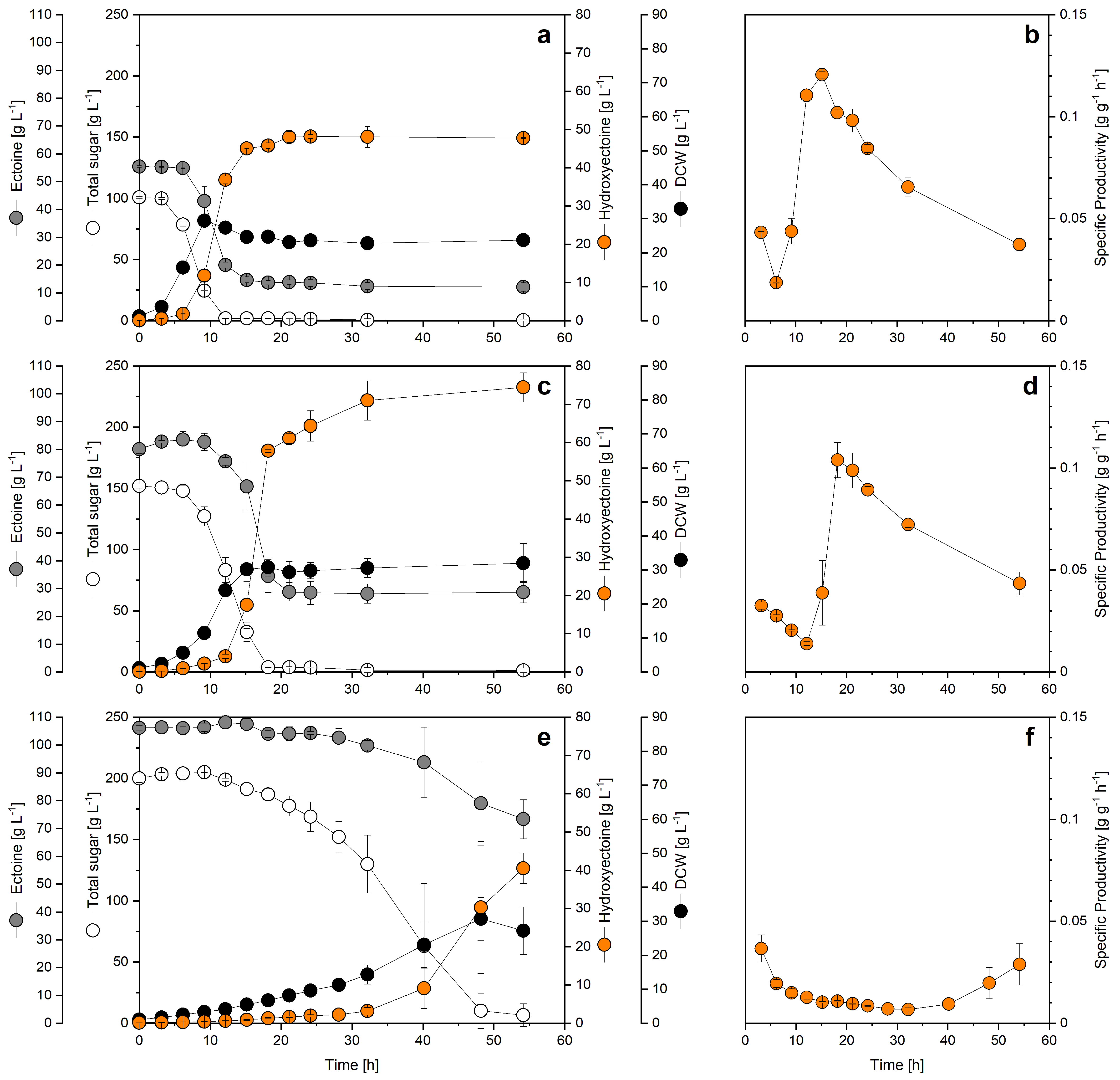


**Figure S6: Production of 5-hydroxyectoine in a batch process in stirred tank bioreactors using *C. glutamicum* *P_tuf_* *ectD^PST^* which episomally expressed codon optimized *ectD* from *Pseudomonas stutzeri* A1501.** The strain was cultivated in batch-mode with initial sucrose and ectoine concentrations of 100 g L^-1^ and 50 g L^-1^ (a, b), 150 g L^-1^ and 75 g L^-1^ (c, d) and 200 g L^-1^ and 100 g L^-1^ (e, f) at 37°C. n=2


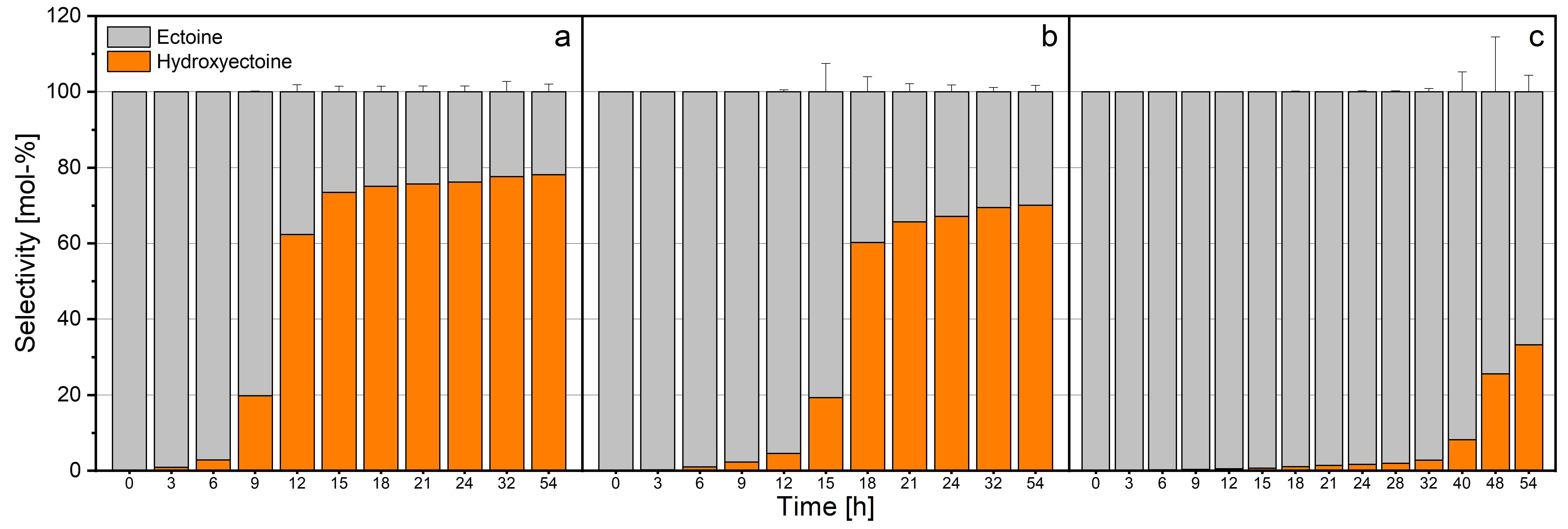


**Figure S7: Selectivity of 5-hydroxyectoine production in a batch process using *C. glutamicum* *P_tuf_* *ectD^PST^*.** The *C. glutamicum* type strain ATCC 13032 episomally expressed codon-optimized *ectD* from *Pseudomonas stutzeri* A1501. It was cultivated with initial sucrose and ectoine concentrations of 100 g L^-1^ and 50 g L^-1^ (a), 150 g L^-1^ and 75 g L^-1^ (b), and 200 g L^-1^ and 100 g L^-1^ (c) at 37°C. n=2

**References**

1. Letunic I, Bork P: **Interactive Tree Of Life (iTOL) v5: an online tool for phylogenetic tree display and annotation.** *Nucleic Acids Res* 2021, **49:**W293-W296.

2. Widderich N, Pittelkow M, Höppner A, Mulnaes D, Buckel W, Gohlke H, Smits SHJ, Bremer E: **Molecular Dynamics Simulations and Structure-Guided Mutagenesis Provide Insight into the Architecture of the Catalytic Core of the Ectoine Hydroxylase.** *J Mol Biol* 2014, **426:**586-600.
