# Supplementary material for: High-efficiency production of 5-hydroxyectoine using metabolically engineered Corynebacterium glutamicum
Source: Microb Cell Fact. 2022 Dec 28;21:274. doi: 10.1186/s12934-022-02003-z (PMC9798599; doi:10.1186/s12934-022-02003-z)
Supplement: Supplementary file 2 — Additional file 2: Table S1. Native (original) and codon-optimized sequences of the used ectD genes. [file 12934_2022_2003_MOESM2_ESM.docx]

**Additional file 2 to**

**High-efficiency production of 5-hydroxyectoine using**

**metabolically engineered Corynebacterium glutamicum**

*Microbial Cell Factories*

Lukas Jungmann, Sarah Lisa Hoffmann, Caroline Lang, Raphaela De Agazio, Judith Becker, Michael Kohlstedt, and Christoph Wittmann*

Institute of Systems Biotechnology, Campus A1.5, Saarland University, Saarbrücken, Germany

*Corresponding author: Phone: +49 681 302 71970, FAX: +49 681 302 71972, e‑mail: [christoph.wittmann@uni-saarland.de](mailto:christoph.wittmann@uni-saarland.de)

**Table S1: Native (original) and codon-optimized sequences of the used ectD genes**

***Pseudomonas stutzeri* A1501 *ectD***

Optimized 1 ATGCAGGCAGATCTGTACCCATCCCGTCAAGAAGATCAGCCATCCTGGCAAGAACGCCTG

Original 1 ATGCAAGCCGACCTGTATCCCTCGCGCCAGGAAGACCAGCCCAGCTGGCAGGAACGCCTG

Optimized 61 GATCCAGTGGTGTACCGCTCCGATCTGGAAAACGCACCAATCGCAGCCGAACTGGTGGAA

Original 61 GATCCGGTCGTCTACCGCAGCGACCTGGAGAATGCGCCGATCGCGGCAGAGCTGGTCGAA

Optimized 121 CGCTTCGAACGCGACGGCTACCTGGTGATCCCAAACCTGTTCTCCGCAGATGAAGTGGCA

Original 121 CGCTTCGAACGCGACGGCTACCTGGTCATCCCCAATCTGTTCAGCGCCGACGAAGTCGCG

Optimized 181 CTGTTTCGCGCAGAACTGGAACGCATGCGCCAGGATCCAGCAGTGGCAGGCTCCGGCAAG

Original 181 CTGTTTCGCGCCGAACTCGAGCGCATGCGCCAGGACCCGGCCGTCGCCGGTTCCGGCAAG

Optimized 241 ACCATCAAAGAACCAGATTCCGGTGCAATCCGCTCCGTGTTCGCAATCCACAAGGATAAC

Original 241 ACCATCAAGGAACCCGACAGCGGTGCGATCCGCTCGGTGTTCGCCATCCACAAGGACAAC

Optimized 301 GAACTGTTCGCTCGCGTGGCAGCAGATGAACGCACCGCAGGTATCGCACGCTTCATCCTG

Original 301 GAGCTGTTCGCCCGCGTCGCAGCCGACGAGCGCACCGCCGGCATCGCCCGCTTCATCCTT

Optimized 361 GGTGGCGATCTGTACGTGCACCAGTCCCGCATGAACTTCAAGCCAGGCTTCACCGGCAAA

Original 361 GGCGGCGACCTGTACGTGCATCAGTCGCGAATGAACTTCAAGCCCGGCTTCACCGGCAAG

Optimized 421 GAATTCTACTGGCACTCCGATTTCGAAACCTGGCACATCGAAGATGGCATGCCACGCATG

Original 421 GAGTTCTACTGGCACTCGGATTTCGAGACCTGGCACATCGAGGACGGCATGCCGCGCATG

Optimized 481 CGCTGCCTGTCCTGCTCCATCCTGCTGACCGATAACGAACCACACAACGGTCCACTGATG

Original 481 CGCTGCCTGTCCTGCTCGATCCTCTTGACCGACAACGAGCCGCACAACGGCCCGCTGATG

Optimized 541 CTGATGCCAGGCTCCCACAAGCACTACGTGCGCTGCGTTGGCGCAACCCCAGAAAACCAC

Original 541 CTGATGCCCGGCTCGCACAAGCACTACGTGCGCTGCGTCGGAGCCACACCGGAAAATCAC

Optimized 601 TACGAAAAGTCCCTGCGCAAGCAAGAAATCGGCATCCCTGATCAGAACTCCCTGTCCGAA

Original 601 TACGAGAAGTCCCTGCGCAAGCAGGAGATCGGCATCCCCGACCAGAACAGCCTGAGCGAG

Optimized 661 CTGGCATCCCGCTTCGGCATCGATTGCGCAACCGGTCCAGCAGGCTCCGTGGTGTTCTTC

Original 661 CTGGCCAGCCGCTTCGGCATCGACTGCGCCACCGGCCCCGCCGGCAGCGTGGTGTTCTTC

Optimized 721 GATTGCAACACCATGCACGGCTCCAACGGCAACATCACCCCATCCGCACGCTCCAACCTG

Original 721 GACTGCAACACCATGCACGGCTCCAACGGCAACATCACGCCCAGCGCGCGTAGCAATCTG

Optimized 781 TTCTACGTGTACAACCACGTGGATAACGCAGTGCAGGCACCATTCTGCGAACAGAAGCCA

Original 781 TTCTACGTCTACAACCACGTGGATAATGCCGTGCAGGCTCCGTTCTGCGAGCAGAAACCG

Optimized 841 CGCCCAGCATTCGTGGCCGAACGCGAAAACTTCAAGCCTCTGGATATTCGCCCACAGCAG

Original 841 CGCCCGGCCTTTGTCGCCGAACGCGAGAATTTCAAGCCGCTGGACATTCGGCCGCAACAG

Optimized 901 TACCTGTAA

Original 901 TATCTCTGA

***Mycobacterium smegmatis* ATCC 19420 *ectD***

Optimized 1 ATGACCACCACCCAGTTCACCCAGACCACCCCAACCGCAGAAACCAGGCACGATCCATAC

Original 1 ATGACCACCACGCAGTTCACCCAGACGACACCAACCGCCGAGACGCGCCACGACCCGTAC

Optimized 61 CCAACCCGCCTGTCCCACGCAATCGAACCAATCCCACGCGCAGACCCAACCGTGTGGGGC

Original 61 CCGACGCGCCTGTCCCACGCGATCGAACCGATCCCGCGTGCCGACCCCACGGTGTGGGGC

Optimized 121 TCCGAAGCCGATGGCCCACTGAACCAGAAGAACCTGGACCACTTCTCCTCCCAGGGCTAC

Original 121 AGCGAGGCCGACGGCCCGCTCAACCAGAAGAACCTGGACCACTTCTCGTCGCAGGGCTAC

Optimized 181 CTGGTGCGCCACGGCACCGTGACCAACGACTGGCTGCCACACCTGCGCCAGGAAATGGAC

Original 181 CTGGTGCGCCACGGCACCGTCACCAACGACTGGCTGCCGCACCTGCGCCAGGAAATGGAC

Optimized 241 CGCATCGCCGCAGACCTGGACGACGATGATCCACGCGTGATCCGCGAACCAGGCGGCTCC

Original 241 CGCATCGCCGCCGATCTCGACGACGACGATCCGCGCGTGATCCGCGAGCCGGGCGGGTCG

Optimized 301 ATCCGCTCCATCTTCGAACCACACCTGCTGTCCGATCTGGTGGCACAGGTGGTGCGCCTG

Original 301 ATCCGCTCGATCTTCGAACCGCACCTGCTCAGCGACCTTGTCGCGCAGGTGGTCCGGCTC

Optimized 361 GATACCGTGCTGCCAGTGGCACGCCAGCTGCTGGGCTCCGATGTGTACATCCACCAGGCA

Original 361 GACACCGTCCTGCCGGTGGCACGCCAGCTGCTCGGTAGCGACGTCTACATCCACCAGGCG

Optimized 421 CGCATCAACATGATGCCAGGCTTCACCGGCACCGGCTTCTACTGGCACTCCGACTTCGAA

Original 421 CGGATCAACATGATGCCCGGGTTCACCGGGACGGGTTTCTACTGGCACTCGGATTTCGAG

Optimized 481 ACCTGGCACGCAGAAGACGGCATGCCAGCAATCCGCGCCGTGTCCTGCTCCATCGCACTG

Original 481 ACGTGGCACGCCGAGGACGGCATGCCGGCCATCCGGGCCGTGTCGTGTTCGATCGCGCTG

Optimized 541 ACCCGCAACTACCCATACAACGGCTCCCTGATGGTGATCCCAGGCTCCCACCAGACCTTC

Original 541 ACCCGCAACTACCCGTACAACGGGTCGCTGATGGTGATCCCCGGCTCGCACCAGACGTTC

Optimized 601 TACCCATGCGTGGGCGAAACTCCTCAGGATAACCACGATACCTCCCTGGTGGCCCAGACC

Original 601 TATCCGTGCGTGGGGGAGACCCCGCAGGACAACCACGACACGTCGCTGGTGGCCCAGACG

Optimized 661 GTGGGCGTGCCAGATGAAACCACCCTGACCAAGGCAGTGGACCACGCAGGCATCGATCAG

Original 661 GTCGGCGTCCCCGACGAGACGACGCTGACCAAGGCCGTCGACCACGCCGGCATCGATCAG

Optimized 721 TTCACCGGCGCAGCAGGCTCCGCACTGTGGTTCGATGCCAACCTGCTGCACGGCTCCGGC

Original 721 TTCACGGGAGCCGCCGGGTCGGCGCTGTGGTTCGACGCCAACCTGCTGCACGGGTCCGGG

Optimized 781 TCCAACATCACCCCACTGCCACGCTCCAACGTGTTCCTGGTGTTCAACTCCGTGGACAAC

Original 781 TCCAACATCACACCGCTTCCGCGGTCCAATGTGTTCCTGGTTTTCAACTCGGTCGACAAC

Optimized 841 GCACTGGAAGAACCATTCGCCGCACCACGCCGCCGCCCAGAATACCTGGCAGCACGCCGC

Original 841 GCGCTCGAGGAGCCTTTCGCGGCGCCACGACGCAGGCCGGAATACCTGGCAGCCCGTCGG

Optimized 901 GTGGCCCCAGTGACTTAA

Original 901 GTCGCACCCGTCACATAG

***Streptomyces coelicolor* A3(2) *ectD***

Optimized 1 GTGGCAACCCCACGCCAGGATCCAGTGGTGTGGGGCTCCCCAGACGCCCCAGGTCCAGTG

Original 1 GTGGCCACCCCTCGGCAGGACCCGGTCGTCTGGGGCTCCCCGGACGCGCCCGGCCCCGTC

Optimized 61 TCCGCCGGTGACCTGCAGGCACTGGACCGCGACGGCTTCCTGGCAATCGATCAGCTGATC

Original 61 TCTGCGGGCGACCTCCAGGCGCTGGACCGCGACGGCTTCCTCGCCATCGACCAGCTGATC

Optimized 121 ACCCCAGACGAAGTGGGCGAATACCAGCGCGAACTGGAACGCCTGACCACCGACCCAGCA

Original 121 ACCCCGGACGAGGTCGGCGAGTACCAGCGTGAGCTGGAGCGCCTCACCACCGACCCGGCC

Optimized 181 ATCCGCGCCGATGAACGCTCCATCGTGGAACCACAGTCCAAGGAAATCCGCTCCGTGTTC

Original 181 ATCCGCGCGGACGAGCGCTCGATCGTCGAGCCGCAGTCCAAGGAGATCCGGTCGGTCTTC

Optimized 241 GAAGTGCACAAGATCTCCGAAGTGTTCGCCAAGCTGGTGCGCGATGAACGCGTGGTGGGC

Original 241 GAGGTGCACAAGATCAGTGAGGTCTTCGCCAAGCTGGTGCGCGACGAGCGCGTGGTCGGG

Optimized 301 CGCGCACGTCAGATCCTGGGCTCCGATGTGTACGTGCACCAGTCCCGCATCAACGTGAAG

Original 301 CGGGCGCGGCAGATCCTCGGCTCGGACGTGTACGTCCACCAGTCGCGGATCAACGTCAAG

Optimized 361 CCAGGCTTCGGCGCATCCGGCTTCTACTGGCACTCCGACTTCGAAACCTGGCACGCCGAA

Original 361 CCGGGCTTCGGGGCCAGCGGTTTCTACTGGCACTCGGACTTCGAGACCTGGCACGCCGAG

Optimized 421 GATGGCCTGCCAAACATGCGCACCATCTCCGTGTCCATCGCACTGACCGAAAACTACGAT

Original 421 GACGGCCTGCCCAACATGCGCACGATCTCGGTCTCGATCGCGCTCACCGAGAACTACGAC

Optimized 481 ACCAACGGCGGCCTGATGATCATGCCAGGCTCCCACAAGACCTTCCTGGGCTGCGCCGGC

Original 481 ACCAACGGCGGTCTGATGATCATGCCGGGGTCGCACAAGACGTTCCTCGGGTGCGCGGGG

Optimized 541 GCCACCCCAAAGGATAACTACAAGAAGTCCCTGCAGATGCAGGATGCAGGCACCCCATCC

Original 541 GCCACGCCGAAGGACAACTACAAGAAGTCCCTGCAGATGCAGGACGCGGGGACGCCGTCC

Optimized 601 GATGAAGGCCTGACCAAGATGGCATCCGAATACGGCATCAAGCTGTTCACCGGCAAGGCA

Original 601 GACGAGGGCCTGACGAAGATGGCCTCGGAGTACGGCATCAAGCTGTTCACCGGCAAGGCC

Optimized 661 GGCTCCGCCACCTGGTTCGACTGCAACTGCATGCACGGCTCCGGCGACAACATCACCCCA

Original 661 GGTTCGGCGACCTGGTTCGACTGCAACTGCATGCACGGGTCGGGCGACAACATCACGCCG

Optimized 721 TTCCCACGCTCCAACGTGTTCATCGTGTTCAACTCCGTGGAAAACACCGCAGTGGAACCA

Original 721 TTCCCGCGCAGCAACGTGTTCATCGTGTTCAACAGCGTGGAGAACACGGCGGTCGAGCCG

Optimized 781 TTCGCAGCACCAATCCGCCGCCCAGAATTCATCGGCGCCCGCGACTTCACCCCAGTGAAG

Original 781 TTCGCGGCTCCGATCCGGCGGCCGGAGTTCATCGGGGCGCGGGACTTCACGCCGGTGAAG

Optimized 841 TAA

Original 841 TGA

***Halomonas elongata* ATCC 33173 *ectD***

Optimized 1 ATGTCCGTGCAGACCTCCTCCAACCGCCCACTGCCACAGGCAAACCTGCACATCGCAACC

Original 1 ATGTCAGTGCAGACATCGTCCAACCGACCGCTGCCACAAGCGAACCTGCATATCGCCACG

Optimized 61 GAAACTCCTGAAGCCGATTCCCGCATCCGCTCCGCACCACGCCCAGGTCAGGACCCATAC

Original 61 GAGACACCCGAGGCCGACAGCCGGATCCGTAGCGCGCCGCGTCCGGGGCAGGATCCCTAT

Optimized 121 CCAACCCGCCTGTCCGAACCACTGGACCTGCCATGGCTGAACCGCCGCGAACCAGTGGTG

Original 121 CCGACCCGACTGAGCGAGCCGCTGGATCTTCCCTGGCTCAATCGCCGCGAGCCGGTGGTC

Optimized 181 AAGGGCGAAGAAGCCGATGGCCCACTGTCCGCAGCCCAGCTGGATACCTTCGAACGCCAG

Original 181 AAGGGAGAGGAGGCCGATGGGCCGCTCTCGGCCGCGCAGCTCGATACCTTCGAGCGCCAG

Optimized 241 GGCTTCATCTTCGAACCAGACTTCCTGAAGGGCGAAGAGCTGGAAGCACTGCGCCACGAA

Original 241 GGCTTCATCTTCGAGCCGGACTTCCTGAAAGGCGAGGAACTCGAGGCGTTGCGCCACGAA

Optimized 301 CTGAACGCACTGCTGGCCCGCGATGACTTCCGCGGCCGTGACTTCGCCATCACCGAACCA

Original 301 CTCAACGCCCTGCTGGCCCGGGATGACTTCCGCGGACGAGACTTCGCCATCACCGAGCCG

Optimized 361 CAGGGCAACGAAATCCGCTCCCTGTTCGCAGTGCACTACCTGTCCCGCGTGTTCTCCCGC

Original 361 CAGGGCAACGAGATCCGCTCGCTGTTCGCGGTGCACTACCTGTCGCGAGTCTTCAGCCGC

Optimized 421 CTGGCAAACGATGAACGCCTGATGGGCCGCGCACGCCAGATCCTGGGCGGTGAACCATAC

Original 421 CTGGCCAACGACGAACGCCTGATGGGTCGCGCCCGGCAGATTCTCGGCGGCGAGCCCTAT

Optimized 481 GTGCACCAGTCCCGCATCAACTACAAGCCAGGCTTCGAAGGCAAGGGCTTCAACTGGCAC

Original 481 GTCCATCAGTCGCGCATCAACTACAAGCCCGGCTTCGAGGGCAAGGGCTTCAATTGGCAT

Optimized 541 TCCGACTTCGAAACCTGGCACGCAGAAGATGGCATGCCAGCAATGCACGCCGTGTCCGCA

Original 541 TCCGATTTTGAAACCTGGCACGCCGAGGATGGCATGCCCGCCATGCATGCGGTGAGTGCG

Optimized 601 TCCATCGTGCTGACCGACAACCACACCTTCAACGGCCCACTGATGCTGGTGCCAGGCTCC

Original 601 TCCATCGTGCTGACCGACAACCACACCTTCAACGGGCCGCTGATGCTGGTGCCCGGCTCA

Optimized 661 CACCGCGTGTTCGTGCCATGCCTGGGCGAAACTCCTGAGGACCACCACCGCCAGTCCCTG

Original 661 CACCGGGTATTCGTGCCGTGCCTGGGTGAAACGCCGGAGGATCATCACCGGCAGTCGCTC

Optimized 721 AAGACCCAGGAATTCGGCGTGCCATCCCGCCAGGCACTGCGCGAACTGATCGATCGCCAC

Original 721 AAGACCCAGGAATTCGGCGTGCCGAGCCGCCAGGCGCTGCGCGAGTTGATCGACCGACAT

Optimized 781 GGCATCGAAGCACCAACCGGCGCCGCCGGTGGCTTGTTGCTGTTCGACTGCAACACCCTG

Original 781 GGTATCGAAGCGCCCACCGGCGCGGCGGGTGGCCTGCTGCTGTTCGACTGCAATACCCTG

Optimized 841 CACGGCTCCAACGCCAACATGTCCCCAGATCCACGCTCCAACGCATTCTTCGTGTACAAC

Original 841 CACGGCTCCAACGCCAACATGTCGCCGGATCCGCGCAGCAACGCCTTTTTCGTCTACAAC

Optimized 901 CGCCGCGATAACCGCTGCGTGGAACCA

Original 901 CGTCGTGACAACCGCTGCGTCGAACCT

***Virgibacillus salexigens ATCC* 700290 *ectD***

Optimized 1 ATGGAAGACCTGTACCCATCCCGCCAGAACAACCAGCCAAAGATCCTGAAGCGCAAGGAT

Original 1 ATGGAAGATCTTTATCCTTCAAGACAAAATAATCAACCGAAGATTTTGAAAAGGAAGGAT

Optimized 61 CCAGTGATCTACACCGACCGCTCCAAGGATAACCAGGCCCCAATCACCAAGGAACAGCTG

Original 61 CCTGTAATCTATACTGATCGATCCAAAGATAATCAAGCCCCTATTACAAAAGAACAGCTT

Optimized 121 GACTCCTACGAAAAGAACGGCTTCCTGCAGATCAAGAACTTCTTCTCCGAAGATGAAGTG

Original 121 GATTCCTATGAGAAAAATGGATTTCTGCAGATTAAAAACTTTTTCTCTGAAGACGAAGTG

Optimized 181 ATCGACATGCAGAAGGCCATCTTCGAACTGCAGGATTCCATCAAGGACGTGGCATCCGAC

Original 181 ATAGATATGCAGAAAGCGATTTTTGAACTGCAGGATTCAATCAAAGATGTGGCTTCCGAT

Optimized 241 AAGGTGATCCGCGAACCAGAATCCAACGATATCCGCTCCATCTTCCACGTGCACCAGGAC

Original 241 AAAGTAATACGCGAACCGGAAAGCAATGACATCCGTTCTATTTTTCACGTTCATCAGGAT

Optimized 301 GACAACTACTTCCAGGATGTGGCAAACGACAAGCGCATCCTGGACATCGTGCGCCACCTG

Original 301 GATAACTATTTCCAAGATGTGGCAAATGACAAACGCATTCTTGATATTGTTCGTCACCTT

Optimized 361 CTGGGCTCCGATGTGTACGTGCACCAGTCCCGCATCAACTACAAGCCAGGCTTCAAGGGC

Original 361 TTGGGAAGCGATGTGTATGTACATCAATCCCGAATTAATTATAAACCGGGATTTAAAGGA

Optimized 421 AAGGAATTCGACTGGCACTCCGACTTCGAAACCTGGCACGTGGAAGACGGCATGCCACGC

Original 421 AAGGAGTTTGACTGGCATTCTGATTTCGAGACATGGCATGTAGAAGATGGAATGCCACGG

Optimized 481 ATGCGCTGCATCTCCGTGTCCATCGCCCTGTCCGACAACTACTCCTTCAACGGCCCACTG

Original 481 ATGCGATGCATCAGTGTATCTATTGCTCTATCAGATAACTATTCGTTTAACGGACCTTTG

Optimized 541 ATGCTGATCCCAGGCTCCCACAACTACTTCGTGTCCTGCGTGGGCGAAACTCCTGATAAC

Original 541 ATGCTTATACCAGGGTCCCATAACTATTTTGTGAGTTGTGTAGGTGAAACACCTGATAAT

Optimized 601 AACTACAAGGAATCCCTGAAGAAGCAGAAGCTGGGCGTGCCAGACGAAGAATCCCTGCGC

Original 601 AACTATAAAGAATCATTGAAGAAGCAGAAGCTGGGCGTACCTGATGAAGAAAGCCTTCGT

Optimized 661 GAACTGACCCGCATCGGCGGCGGTATCTCCGTGCCAACCGGCAAGGCAGGCTCCGTGACC

Original 661 GAACTAACAAGAATAGGCGGGGGTATCTCTGTTCCAACTGGTAAGGCAGGATCTGTTACA

Optimized 721 CTGTTCGAATGCAACACCATGCACGGCTCCACCTCCAACATCACCCCATACCCACGCAAC

Original 721 TTGTTTGAATGTAACACAATGCATGGTTCAACCAGCAATATTACACCATATCCACGTAAT

Optimized 781 AACCTGTTCATGGTGTACAACTCCGTGAAGAACCGCCTGGTGGAACCATTCTCCGGCGGC

Original 781 AATTTATTCATGGTGTATAATAGTGTTAAAAATCGTTTAGTGGAGCCGTTTTCTGGTGGT

Optimized 841 GAAAAGCGCCCAGAATACATCGCAGTGCGCGAAAAGCAGCCAGTGTACTCCGCAGTGAAC

Original 841 GAAAAGCGTCCGGAATATATAGCAGTACGAGAGAAACAACCAGTGTATAGTGCTGTAAAC

Optimized 901 TAA

Original 901 TAA

***Gracilibacillus sp.* SCU50 *ectD***

Optimized 1 ATGAAGGATCTCTACTCCTCCCGCATCAAGGGCATGCCATCCATCCTGGAACGCAAGGAC

Original 1 ATGAAAGATTTATATTCTTCTAGAATCAAAGGCATGCCTTCTATTTTGGAAAGAAAAGAT

Optimized 61 CCAATCATCCACAACTCCACCGCAGAGGATGGCCCACTCCAGCAGAAGGAACTGGACTTC

Original 61 CCAATAATTCACAACTCAACAGCAGAGGATGGTCCTTTACAACAAAAGGAATTAGATTTT

Optimized 121 TACGAGAAGAACGGCTACCTCTTCAAGCAGAACTTCTTCTCCAAGGAAGAGGTGAAGGTC

Original 121 TACGAGAAAAATGGTTATCTATTTAAACAGAACTTTTTTTCTAAGGAAGAGGTAAAGGTA

Optimized 181 CTCCAGCAGGAACTGAAGCGCAACATGAACGATAACCAGTCCTCCGACGAGCCATACGTG

Original 181 TTACAGCAAGAGTTAAAACGTAATATGAATGACAATCAGTCTTCTGATGAACCTTACGTC

Optimized 241 GTCCGCGAAACCGGCGGCGATGAGATCCGCTCCGTGTTCGATGTCCACAACAACGACGAA

Original 241 GTTAGAGAAACTGGTGGCGACGAAATACGTTCAGTATTTGATGTGCATAACAACGATGAA

Optimized 301 TTCTTCCACAACCTGTCCTCCTACGAACGCATCGTGGAGATCGCCCAGCACCTGCTCGGC

Original 301 TTTTTCCACAATTTATCGTCCTATGAACGTATTGTTGAGATTGCACAACATTTGCTAGGA

Optimized 361 TCCCAGGTCTACATCAACCAGTCCCGCATCAACTTCAAGCCAGGCTTCAAGGGCAAGGGC

Original 361 AGTCAGGTTTACATAAACCAATCACGGATTAATTTTAAACCTGGCTTTAAAGGAAAAGGC

Optimized 421 TTCTACTGGCACTCCGATTTCGAAACCTGGCACATGGAGGACGGCATGCCAAACATGCGC

Original 421 TTTTATTGGCATTCTGATTTTGAAACCTGGCATATGGAAGATGGAATGCCTAATATGCGT

Optimized 481 GCTGTGTCCTGCTCCATCATCCTGACCGACAACTACTCCTACAACGGCCCACTGCTCCTG

Original 481 GCTGTTAGTTGCAGTATTATTTTAACGGACAACTATTCTTACAACGGTCCACTATTATTA

Optimized 541 ATCCCAGGCTCCCACCGCTACTTCGTGCAGTGCGTCGGCGAAACCCCAGAGAACCACTTC

Original 541 ATTCCTGGTTCCCATCGTTACTTTGTACAGTGTGTTGGGGAAACACCTGAAAATCATTTT

Optimized 601 GAACAGTCCCTCAAGATGCAGAACATCGGCGTGCCAGATCACGACTCCATCAACTGGCTC

Original 601 GAACAGTCCTTGAAAATGCAAAATATCGGTGTACCAGATCATGATAGTATTAACTGGTTA

Optimized 661 CTGGAAAAGGGCGGCCGTATCGATTCCGCAACCGGTCCAGCTGGCTCCGTCCTGTTCTTC

Original 661 TTAGAAAAAGGCGGAAGAATAGACAGCGCTACAGGACCGGCTGGGTCAGTATTATTCTTT

Optimized 721 GAGTCCAACACCATGCACGGCTCCAACTCCAACATCTCCCCACTCCCACGCTCCAACGTG

Original 721 GAATCTAATACCATGCATGGTTCTAATAGTAATATTTCGCCACTGCCACGTAGTAATGTA

Optimized 781 TTCTTCGTCTTCAACTCCATCGAAAACAAGCTCGTGGAACCATTCGCTGACGTCTCCGCA

Original 781 TTTTTTGTCTTTAACAGTATTGAAAATAAATTGGTGGAACCATTCGCGGATGTTTCTGCA

Optimized 841 CGCCCAGAATTCGTGGCCAACCGCACCAACATCAAGCCAATCGAGCCAAAGCACTTCAAC

Original 841 AGACCAGAGTTTGTTGCAAATCGTACAAATATTAAACCGATCGAACCGAAACATTTTAAT

Optimized 901 CTCAACCGTCGCAAGGTCTACCTGCACCAGTAA

Original 901 CTTAATAGGAGAAAAGTGTATTTACATCAATAA

***Acidiphilium cryptum* JF5 *ectD***

Optimized 1 ATGGATGATTTGTACCCATCCCGCCGCGAGCCAACCCCATCCTTGCTCCCACGTCACGAC

Original 1 ATGGACGATCTCTATCCGTCGCGCCGCGAACCCACCCCGTCGCTGCTGCCGCGCCACGAT

Optimized 61 CCAGTTGTCCACGGCCGCTGGGCTCCAGGTGCACCACTCTCCGACGAACAGACCCGCTTC

Original 61 CCGGTGGTGCATGGGCGCTGGGCGCCCGGCGCGCCGCTGAGCGACGAGCAGACGCGGTTC

Optimized 121 TACGATACCAACGGCTACCTGGTGCTCGAGAACGTCTTCGATCCAGCCGAAATCGCTCTG

Original 121 TACGACACCAACGGCTATCTCGTGCTGGAGAACGTGTTCGACCCCGCCGAGATCGCGCTG

Optimized 181 CTCCAGTCCGGCTCCATGGACCTGCTCGCAAACCCAGCCGGCCTGGATCGCGAAACCATC

Original 181 CTGCAGTCGGGGTCGATGGATCTGCTGGCCAACCCCGCCGGGCTCGACCGCGAGACCATC

Optimized 241 ATCACCGAGCGCGGCTCCGATGAAGTGCGTTCCATCTTCGCTATCCACGCACAGAACGAA

Original 241 ATCACCGAGCGCGGCAGCGACGAGGTGCGCTCGATCTTCGCGATCCACGCGCAGAACGAA

Optimized 301 TTGCTGGGCCGCCTGGCAGCTGATTCCCGCATCGCCGGCGTGGCTCGCTTCCTGCTCGAT

Original 301 TTGCTCGGCCGTCTCGCCGCGGATTCGCGGATTGCCGGGGTGGCGCGCTTCCTGCTGGAT

Optimized 361 GACGATGTCTACATCCACCAGTCCCGCCTCAACTACAAGCCAGGCTTCGATGGCAAGGAG

Original 361 GACGACGTCTACATCCATCAGTCGCGGCTCAACTACAAGCCGGGCTTCGATGGCAAGGAG

Optimized 421 TTCTACTGGCACTCCGACTTCGAGACCTGGCACGTCGAAGATGGCATGCCACGCATGCGC

Original 421 TTCTACTGGCACTCCGATTTCGAGACCTGGCATGTCGAGGATGGCATGCCGCGGATGCGC

Optimized 481 GCACTGTCCATGTCCATCCTGCTCGCTGAAAACACCGCAAACAACGGCCCACTGATGGTG

Original 481 GCGTTGTCGATGTCGATCCTGCTGGCGGAGAATACCGCGAATAACGGGCCGCTGATGGTC

Optimized 541 ATCCCAGGCTCCCACCGCAAGTACCTCACCTGCGTCGGCGAAACCCCAGAGAACCACTAC

Original 541 ATCCCCGGCTCGCACCGCAAATATCTCACCTGCGTTGGCGAGACGCCGGAGAACCATTAC

Optimized 601 CGCTCCTCCCTGAAGAAGCAGGAGTACGGCGTGCCAGACCGCGAAATGCTGACCGCCCTC

Original 601 CGCTCCTCGCTGAAGAAGCAGGAATACGGCGTGCCCGACCGCGAGATGCTGACCGCGCTC

Optimized 661 GCTTCCGATCACGGTATCGTGGCACCAACCGGCAAGGCTGGTACCGTGGTCCTCTTCGAC

Original 661 GCCAGCGATCACGGCATCGTCGCGCCGACGGGCAAGGCCGGCACCGTGGTGCTGTTCGAC

Optimized 721 TGCAACACCATGCACGGCTCCAACGGCAACATCACCCCATTCCCACGCTCCAACGCCTTC

Original 721 TGCAACACGATGCACGGCTCGAACGGCAACATCACGCCGTTCCCGCGCTCGAACGCGTTC

Optimized 781 TTCGTGTTCAACGCAAAGGCCAACTCCCTGGTCGAGCCATTCGGTCCAAAGTCCCGCCGC

Original 781 TTCGTGTTCAACGCGAAGGCGAACAGCCTGGTCGAGCCGTTCGGCCCGAAATCGCGGCGG

Optimized 841 CCAGACTTCATCGCAGATCGCGCCTTCACCACCGTGGACATCGTGAAGGGTCCACTGGTG

Original 841 CCGGACTTCATCGCCGACCGCGCGTTCACGACCGTCGACATCGTGAAGGGCCCGCTGGTG

Optimized 901 CGTCGCGAACGTGCTGCATAA

Original 901 CGGCGCGAGCGCGCGGCATGA

***Alkalihalobacillus clausii* 7520-2 *ectD***

Optimized 1 ATGCAGGAAAAGGTGGACCTGTACCCATCCCGCGTCAAGGAAACCGCCACCATCACCGAA

Original 1 ATGCAAGAAAAGGTAGATCTCTATCCTTCACGCGTTAAGGAGACCGCAACCATAACGGAA

Optimized 61 CGCAAGGACCCAATCGTGTACTCCCGCGAGAAGGGTCCACTGGACCGTGAAGAGGTGGCT

Original 61 AGAAAAGATCCAATCGTTTATTCAAGGGAAAAAGGACCGCTCGATCGTGAGGAAGTGGCT

Optimized 121 TTCTACGAAGCAAACGGCTACGTCATGCTGGAGCGCCTCTTCCAGGAAGATGAGGTGCAG

Original 121 TTTTATGAGGCAAATGGGTATGTAATGTTAGAACGGCTTTTTCAAGAAGACGAAGTGCAA

Optimized 181 ATCATGGCAAACCAGCTGGAAGAGGTCATGAAGCAGAACCAGGAACGCGATTCCGACGAA

Original 181 ATCATGGCTAATCAATTGGAAGAGGTGATGAAACAGAACCAAGAGCGGGACAGTGATGAA

Optimized 241 GTCATCAAGGAGCCAGATTCCAACGAAATCCGCTCCGTGTTCGAGGTCCACAAGGACAAC

Original 241 GTCATCAAAGAGCCTGATAGCAATGAAATTCGCTCCGTTTTTGAAGTCCACAAAGACAAC

Optimized 301 GGCTTCTTCGAAATGCTGTCCAAGCACGAGCGCATCGTGGCAATCGCCGAACAGCTGCTC

Original 301 GGCTTTTTTGAAATGTTGTCTAAACATGAGCGCATTGTGGCCATTGCAGAACAATTGCTA

Optimized 361 GGCTCCCACGTCTACATCAACCAGTCCCGCATCAACTTCAAGCCAGGCTTCAAGGGCAAG

Original 361 GGCAGCCACGTGTACATCAACCAATCGCGCATTAATTTTAAACCTGGCTTTAAGGGCAAA

Optimized 421 GAGTTCTTCTGGCACTCCGATTTCGAAACCTGGCACGTGGAGGACGGCATGCCAAACATG

Original 421 GAGTTTTTCTGGCATTCTGATTTTGAAACGTGGCATGTGGAAGATGGGATGCCAAACATG

Optimized 481 CGCGCTGTCTCCTGCTCCATCATCCTGACCGACAACTACGAATTCAACGGCCCACTGATG

Original 481 CGGGCTGTCAGCTGCTCGATTATACTTACGGATAACTATGAATTTAACGGGCCTTTAATG

Optimized 541 CTCATCCCAGGCTCCCACAAGTGGTACGTGTCCTGCGCAGGCACCACCCCAGATAACCAC

Original 541 CTTATCCCAGGCTCACATAAATGGTATGTTTCTTGTGCGGGCACAACGCCAGACAACCAT

Optimized 601 TACAAGTCCTCCCTGAAGCAGCAGGTGGCTGGTACCCCAGACCACACCTCTTTGCAGTGG

Original 601 TACAAATCATCGTTAAAGCAACAAGTTGCGGGAACGCCTGACCATACAAGTTTGCAATGG

Optimized 661 CTGACCGAACAGGCTGGCGGCCGTATCGATCGTGCAACCGGCCCAGCTGGCTCCGTGCTG

Original 661 CTGACTGAACAAGCGGGCGGACGGATTGACCGTGCTACTGGCCCAGCCGGATCGGTGCTG

Optimized 721 TTCTTCGAGTGCAACACCATGCACGGCTCCAACGCAAACCTCTCCCCATACCCACGCTCC

Original 721 TTTTTTGAGTGCAATACGATGCATGGCTCCAATGCCAACCTTTCTCCTTACCCACGAAGC

Optimized 781 AACGTGTTCTTCGTCTTCAACTCCATCGAAAACCAGCTGCAGGCACCATACTCCGGCAAG

Original 781 AACGTCTTTTTCGTATTTAACTCAATTGAAAACCAGTTGCAGGCACCTTACTCAGGAAAA

Optimized 841 CAGCCACGTCCAGAGTTCCTCGCTAACCGCGATTCCATCGCACCAATCCAGCCAGTGAAG

Original 841 CAACCAAGGCCTGAATTTTTAGCCAACCGCGACAGCATTGCCCCAATTCAACCTGTAAAA

Optimized 901 GAATCCATCGCACAGCACGTCCACGCCACCAAGTAA

Original 901 GAAAGCATTGCCCAACATGTCCACGCAACGAAATAA

***Hydrocarboniclastica marina* KCTC 62334 *ectD***

Optimized 1 ATGCTCCAGCGCACCGACTTCTACCCATCCCGCCTGGATTCCCCAGCTGACCGTCTCCGT

Original 1 ATGCTACAGCGTACAGACTTTTATCCATCCAGACTGGATTCGCCGGCCGACAGACTTCGT

Optimized 61 CGCACCGCTCCAACCGTGCACGCACAGGGTCCAAAGCGTTGGCAGGGTCCACTGGATGAG

Original 61 CGCACAGCACCCACCGTTCACGCTCAAGGTCCGAAACGATGGCAGGGTCCGCTGGATGAA

Optimized 121 CAGTCCCTCGCACGCTTCGAACGCGATGGCTTCCTGTGGTTCGACGGCTTCTTCTCCCGC

Original 121 CAGAGCCTGGCCCGTTTCGAGCGAGACGGTTTTCTCTGGTTCGATGGTTTCTTCTCCCGC

Optimized 181 GATCGCGTCACCCCATTCCTCGACGAACTGCAGGAGCTCGCTAAGGATACCGCCCTCGCT

Original 181 GACCGGGTCACGCCGTTTCTGGATGAGCTTCAGGAGCTGGCGAAGGACACTGCCCTGGCA

Optimized 241 AACTCCGAGCAGGTGATCAAGGACCCACAGTCCGGTGACCTGCGCTCCGTCTTCGCAATG

Original 241 AATTCAGAACAGGTCATCAAAGACCCGCAAAGTGGCGATCTACGCTCAGTTTTCGCTATG

Optimized 301 CACGAACTGTCCGAGCGCTTCGATCAGCTGACCCGCGACCCACGCATCCTGGGTATGGTG

Original 301 CACGAGCTCTCCGAGCGCTTCGACCAGCTGACCCGGGACCCACGAATACTGGGTATGGTG

Optimized 361 CAGCAGCTGCTCGGCGGCGACGTCTACATCCACCAGTCCCGCATCAACTCCAAGACCGGC

Original 361 CAGCAGCTCCTCGGCGGGGATGTCTACATCCATCAGTCCCGTATAAACAGTAAAACCGGC

Optimized 421 TTCGCTGGCTCCGGCTTCGAATGGCACTCCGACTTCGAAACCTGGCACGCAGAGGATGGC

Original 421 TTTGCGGGTAGCGGTTTCGAGTGGCATTCAGACTTTGAAACCTGGCATGCAGAGGACGGC

Optimized 481 ATGCCAGAAATGCGCGCAGTGTCCGCCTCCCTCATGCTGACCGATAACTCCCCATTCAAC

Original 481 ATGCCAGAAATGCGAGCAGTGAGCGCGTCACTGATGCTGACGGACAACAGTCCCTTCAAT

Optimized 541 GGCCCACTCATGCTGATCCCAGGCTCCCACGACTACTTCGTGCCATGCGTGGGTCGTACC

Original 541 GGCCCGCTCATGCTCATACCCGGCTCCCACGATTATTTCGTGCCTTGCGTCGGCCGCACG

Optimized 601 CCAGCAGATAACTGGAAGCAGTCCCTCAAGGACCAGTCCATCGGCGTGCCAGATCGTTCC

Original 601 CCCGCGGATAACTGGAAACAATCGCTGAAGGATCAGAGCATTGGTGTTCCCGACCGCAGC

Optimized 661 CACATCGCAGCACTGGCTGAACGCGGTGGCATCGAAGCTCCAACCGGTCCAGCTGGCTCC

Original 661 CATATTGCAGCTCTGGCTGAGCGCGGCGGAATCGAGGCACCTACCGGACCAGCAGGCTCT

Optimized 721 CTGCTCCTGTTCGAATGCAACACCCTGCACGCATCCAACTCCAACATGTCCCCATGGCCA

Original 721 CTGCTGTTGTTCGAATGCAACACCCTGCATGCCTCAAACAGCAACATGTCACCCTGGCCC

Optimized 781 CGCGCCAACCTCTTCTTCGTGTACAACTCCGTCGAGAACCAGCTCGTGGAACCATTCTCC

Original 781 CGGGCTAACCTGTTCTTCGTCTACAACAGTGTCGAGAATCAACTGGTCGAGCCCTTCAGC

Optimized 841 GGCTCCGCTGCTCGCCCAGAGTTCCTGGGTGCTCGTAAGAACGTCAAGCCACTGACCATG

Original 841 GGATCGGCAGCACGTCCTGAGTTTCTGGGCGCGCGTAAAAACGTCAAGCCGTTGACCATG

Optimized 901 CAGGAACGCCACGTGGAAGCTGATGAGACCCAGCACGTCTACTCCAACTAA

Original 901 CAAGAACGCCATGTCGAGGCCGATGAAACTCAGCACGTCTACAGCAACTAG

***Methylomicrobium alcaliphilum* DSM 19304 *ectD***

Optimized 1 ATGATCACCGAAAACGCAGCCCAGTCCGAGCAGTCCGAAGATTTCTACCAGTCCCGCAAC

Original 1 ATGATTACCGAGAATGCCGCACAGTCCGAACAAAGTGAAGATTTTTATCAATCACGTAAC

Optimized 61 GGCTCCAAGCCAAAGATCATCCCACGCGTGGACCCAGTGGTCTACGCCCAGACCGCTAAC

Original 61 GGTAGTAAGCCGAAAATAATTCCGCGCGTAGACCCGGTAGTTTATGCGCAAACAGCTAAT

Optimized 121 CCAGGCCTGATCGCCGAGGATCTGCAGGCTCGCTACGAACAGCAGGGCTTCCTGGTGATC

Original 121 CCAGGTCTCATTGCAGAGGACTTGCAAGCACGTTATGAGCAACAAGGTTTTCTTGTTATT

Optimized 181 GATAACGTCTTCAACGAGCGCGAAGTGGATTGCTTCAAGCAGGAGCTGAAGCGCCTCAAC

Original 181 GATAATGTTTTTAATGAGAGGGAGGTCGACTGTTTCAAGCAAGAGCTCAAACGCTTGAAC

Optimized 241 GATGACGAAAAGATCAAGGCCTCCGCTGAGGCAATCACCGAACTGTCCTCCGACGAGCTG

Original 241 GACGATGAAAAGATAAAAGCCTCGGCGGAAGCGATAACTGAATTATCCAGCGACGAACTC

Optimized 301 CGCTCCCTCTTCAAGATCCACGAAGTGTCCCCAGTCTTCAAGCGCCTCGCTGCAGATAAC

Original 301 CGTTCACTATTTAAAATTCATGAAGTCAGTCCGGTTTTTAAAAGGTTAGCTGCCGATAAT

Optimized 361 CGTCTGGCAGGTCTGGCACAGCACCTGCTCAACGACCGTGTGTACATCCACCAGTCCCGC

Original 361 CGATTAGCGGGACTGGCTCAACATCTTTTGAACGACCGGGTTTATATTCATCAGTCGCGC

Optimized 421 CTGAACTACAAGCCAGGCTTCCGCGGCAAGGAATTCTACTGGCACTCCGATTTCGAGACC

Original 421 TTAAACTATAAGCCGGGTTTTCGCGGCAAGGAATTTTACTGGCATTCGGACTTTGAAACT

Optimized 481 TGGCACGTCGAAGACGGCATGCCACGCATGCGCGCTCTGTCCATGTCCATCATCCTCACC

Original 481 TGGCATGTAGAAGACGGTATGCCTAGAATGCGTGCGCTCAGCATGTCCATTATTCTTACC

Optimized 541 GAGAACGATCAGCACAACGGCCCACTGATGCTCGTGCCAGGCTCCCACAAGAAGTTCGTG

Original 541 GAAAACGATCAGCATAACGGGCCTTTGATGTTGGTTCCCGGATCGCATAAAAAATTTGTC

Optimized 601 GTCTGCGAAGAGGAAACCCCAGAAAACCACTACTCCGTCTCCCTGAAGAAGCAGGAGTAC

Original 601 GTTTGCGAAGAGGAAACGCCGGAAAATCATTATTCGGTCTCGTTGAAAAAGCAGGAGTAC

Optimized 661 GGCATCCCATCCGATGAATGCCTGGCTTCCCTCGTGGCAGACGGTGGCATCGTCTCCGCA

Original 661 GGCATACCCAGCGATGAATGCTTGGCTAGCTTGGTTGCCGATGGCGGCATCGTATCGGCC

Optimized 721 AACGGCAAGCCAGGCTCCGTGCTGATCTTCGATTCCAACGTCATGCACGGCTCCAACTCC

Original 721 AATGGAAAACCCGGCAGTGTCTTGATTTTCGACAGTAATGTCATGCACGGTTCGAATAGT

Optimized 781 AACATCACCCCATGGCCACGCTCCAACCTCTTCTTCGTGTACAACGCCATCAACAACCGC

Original 781 AATATCACTCCATGGCCTCGCTCGAATCTCTTTTTCGTCTATAACGCGATCAATAATCGA

Optimized 841 GTCACCTGGCCATTCTGCGGTCTGCTCCCACGTCCAGAGTACCTGTGCTCCCGCAAGAAC

Original 841 GTAACATGGCCGTTTTGCGGTTTATTGCCGCGTCCTGAATATCTTTGCAGTCGCAAGAAT

Optimized 901 ATCCGCGTGATCGAACCACGCCCATTCATCGCCGCTGCAGACCAGCTCATCTACGCCTAA

Original 901 ATACGAGTTATCGAACCGCGGCCTTTTATCGCGGCCGCCGATCAATTGATATATGCTTAG

***Neptunomonas concharum* LHW37 *ectD***

Optimized 1 ATGCAGGATGACTACCCATCCCGCATCGAAACCCGTGCAAAGGTGCAGCCACGTCTGGAC

Original 1 ATGCAAGACGATTACCCCTCACGCATAGAGACCAGAGCCAAGGTGCAACCGCGCCTTGAT

Optimized 61 CCAGTCATCTACCCAGAAGCCGAGACCCCAATCCTCTCCTCCTGCACCCTGTCCACCCAG

Original 61 CCGGTTATTTATCCTGAAGCGGAAACACCCATACTATCTTCCTGTACGTTGTCCACCCAG

Optimized 121 CAGCTCAACGACTTCGAGAAGAACGGCTACCTGCTCCTGCCAGGCCTCTTCAACTCCTCC

Original 121 CAACTTAACGATTTTGAGAAGAATGGCTACCTTTTGCTTCCCGGACTGTTTAATTCGTCG

Optimized 181 GAAGTCAACGAGTTCCACCAGGCAATCACCGCCATCCAGCAGGATGAATCCCTGAAGAAC

Original 181 GAGGTCAATGAGTTTCACCAAGCCATCACTGCTATACAGCAAGATGAGTCTCTCAAAAAC

Optimized 241 TCCCCAGTGGCTATCACCGAACGTGGTAACTCCGAGCTGCGCTCCGTCTTCCAGATCCAC

Original 241 AGCCCTGTTGCCATTACCGAAAGGGGCAATTCAGAGCTACGCTCTGTTTTCCAAATTCAC

Optimized 301 CAGAACCACCCACTCTTCTCCAACGTGGCACGCGATCCACGCATCGCTGACATCGCACGC

Original 301 CAGAATCATCCGCTGTTTTCAAACGTTGCCCGCGACCCACGCATTGCTGATATTGCACGG

Optimized 361 CGCATCCTGGGCGGCGATGTCTACATCCACCAGTCCCGCCTCAACTTCAAGCCAGGCTTC

Original 361 CGCATTCTTGGCGGTGATGTTTATATTCATCAGTCACGTCTTAACTTCAAGCCAGGCTTT

Optimized 421 AAGGGCAAGGAGTTCTACTGGCACTCCGATTTCGAAACCTGGCACGTGGAGGATGGTATG

Original 421 AAAGGCAAGGAGTTTTATTGGCACTCGGATTTTGAAACCTGGCATGTAGAAGATGGCATG

Optimized 481 CCACGTATGCGTGCCATCTCCTGCTCCATCCTCCTGACCGACAACAACGCCAAGAACGGC

Original 481 CCTCGTATGCGAGCAATCAGCTGCTCCATTCTTTTGACCGATAATAATGCGAAAAATGGC

Optimized 541 GCTCTCATGCTGATGCCAGGCTCCCACAAGGAATTCATCTCCTGCGTCGGCGAAACCCCA

Original 541 GCTTTAATGTTGATGCCAGGCTCTCATAAAGAGTTCATCAGTTGCGTTGGCGAAACGCCT

Optimized 601 GAGAACCACTACGAAACCTCTCTCCAGAAGCAGGAGTACGGCATCCCATCCAACGAATCC

Original 601 GAGAACCACTACGAGACATCATTGCAAAAGCAAGAATATGGCATTCCCAGCAACGAAAGC

Optimized 661 CTCCAGTACCTGTCCGATAAGTACGGTATCGACTGCGCAGAGGCAGCTGCTGGCTCCGTG

Original 661 CTGCAATACCTAAGCGATAAATATGGTATTGATTGCGCGGAAGCCGCCGCAGGCTCTGTG

Optimized 721 CTGTTCTTCGACTGCAACATCATGCACGGCTCCAACTCCAACATCACCCCAGATCCACGC

Original 721 CTGTTTTTTGATTGCAACATCATGCATGGTTCCAACAGTAACATTACGCCGGACCCTCGC

Optimized 781 TCCAACCTGTTCTTCGTGTACAACCACATCGACAACATGGTCTACGAACCATTCTCCCTC

Original 781 AGCAACCTATTCTTTGTCTACAACCATATTGACAACATGGTTTATGAGCCTTTTTCTCTA

Optimized 841 CAGCCACCACGCCCAGAATACATCTGCTCCCGCGAGACCATCAAGACCCTGTAA

Original 841 CAACCTCCACGGCCAGAATATATCTGCTCGCGCGAGACCATAAAAACCCTTTAA

***Leptospirillum ferriphilum* ML-04 *ectD***

Optimized 1 ATGCCATTCCGCCTGGAAGATCGCTACCCAACCCGCAAGAACCCAGAACCAGTCTGGATG

Original 1 ATGCCATTTCGACTGGAAGACCGCTACCCGACCCGCAAAAACCCTGAACCTGTCTGGATG

Optimized 61 GAGCGCCCAGTTCCAGTGCTGTACCCAGGTCACCGTACCCCATCCCCACTGTCCGCAGAA

Original 61 GAGAGGCCGGTTCCCGTGCTGTATCCGGGACACCGGACCCCCTCTCCCCTTTCCGCCGAG

Optimized 121 CAGGCCGGCCAGTTCGATCGCGACGGCTTCCTGGTGCTCCCACGCATCTACTCCGATGAA

Original 121 CAGGCCGGTCAGTTTGACCGGGACGGATTCCTCGTGTTGCCCCGGATCTACTCGGATGAG

Optimized 181 GAGGTGCGCGTCTTCCGTGAAGAGGTGGAACGCCTGCGTCTCGACCCAGAAGTCCGTGCT

Original 181 GAAGTGCGGGTCTTCCGGGAGGAGGTCGAGCGTTTAAGATTGGATCCGGAGGTCAGGGCT

Optimized 241 TCCGAGAAGACCATCCGTGAACCACAGGGTGATGCTGTGCGCTCCGTCTTCGCTATCCAC

Original 241 TCGGAAAAAACCATCCGGGAACCCCAGGGAGACGCTGTGCGTTCGGTGTTCGCGATACAC

Optimized 301 CGCGATAACCCACTCTTCGCACGTGTTGCAGCTGATGAACGTATCGCTGGCGTCGCACGC

Original 301 CGCGACAATCCTCTGTTTGCCCGCGTGGCAGCGGATGAGAGAATCGCGGGAGTCGCGCGC

Optimized 361 TTCCTGCTCGGCGGCGACGTGTACATCCACCAGTCCCGCCTGAACTACAAGCCAGGCTTC

Original 361 TTCCTCCTGGGAGGAGACGTCTATATCCACCAGTCCCGCCTGAACTACAAACCGGGATTC

Optimized 421 AAGGGCAAGGAGTTCTACTGGCACTCCGATTTCGAGACCTGGCACGCTGAAGATGGCATG

Original 421 AAGGGAAAAGAGTTTTACTGGCACTCGGATTTCGAGACATGGCATGCGGAAGACGGCATG

Optimized 481 CCAGACATGCGCGCAGTCTCCTGCTCCATCCTGCTCACCGACAACACCACCTCTAACGGC

Original 481 CCCGACATGCGGGCGGTAAGCTGTTCCATCCTCCTGACCGACAACACCACCTCTAACGGC

Optimized 541 CCACTGATGCTCATCCCAGGCTCCCACCGCCACTTCATCCACTGCGTCGGCCAGACCCCA

Original 541 CCCCTGATGCTGATTCCCGGCTCCCATCGCCACTTCATCCATTGCGTCGGGCAGACCCCC

Optimized 601 GAGAACCACTACCGCGAATCCCTCCGCAAGCAGGAGTACGGCGTGCCAGATCAGGCATCC

Original 601 GAGAACCATTACCGGGAATCCCTCCGAAAACAGGAGTACGGCGTTCCGGACCAGGCAAGC

Optimized 661 CTGGAAAAGCTCGCTGCACTGTACGGTATCACCGAGGTGACCGCACCAGCTGGCTCCGCA

Original 661 CTCGAAAAACTCGCCGCTCTTTATGGAATCACGGAAGTGACGGCACCGGCGGGGAGCGCG

Optimized 721 GTCTTCTTCGACTGCAACATCATGCACGGCTCCAACTCCAACATCACCCCATTCCCACGC

Original 721 GTTTTCTTCGACTGCAACATCATGCACGGCTCCAACAGCAACATCACCCCTTTTCCGCGC

Optimized 781 ACCAACCTCTTCTACGTGTACAACCACACCGGTAACGCTGTCCAGGAAGGCCTGCGTACC

Original 781 ACAAATCTTTTTTATGTCTACAACCATACGGGGAATGCCGTGCAGGAAGGACTTCGGACG

Optimized 841 CACCCACCACGCCCAGACTTCGTGGCTGAATCCAAGAACTTCACCCCACTGGCAATCCGC

Original 841 CACCCTCCGAGACCGGATTTCGTCGCCGAATCCAAAAACTTCACTCCTCTCGCGATTCGT

Optimized 901 CCAGAGCGCTTCGCCTAA

Original 901 CCGGAGCGCTTTGCCTGA

***Candidatus Nitrosopumilus sp.* AR2 *ectD***

Optimized 1 ATGGCAGAGAACAAGTTCTACCTGGATTCCTACCCTACCCGCACCTCTTCCGAATCCAAG

Original 1 ATGGCTGAAAATAAGTTTTATTTGGATTCTTATCCTACACGAACAAGTTCTGAATCAAAA

Optimized 61 ATCATCCAGCGCACCAACTCCGTGGTCTACTGCGATTCCTACGACGAACTGAACAAGGAG

Original 61 ATTATTCAAAGAACGAACTCTGTTGTATATTGTGATTCTTATGATGAATTAAATAAAGAA

Optimized 121 CAGGTGGACTTCTTCGAAAAGAACGGCTACCTGATCTTCGAAAACCTCTTCTCCTCCGAT

Original 121 CAAGTAGATTTTTTTGAAAAAAATGGTTATCTAATATTTGAAAATTTATTTTCTTCTGAT

Optimized 181 GAGATCACCAAGCTCTTCGACGAACTGACCTCTCTCTCCCAGGATGAGATCAAGAAGGAC

Original 181 GAGATAACTAAACTGTTTGATGAGCTTACCTCTCTTTCACAAGATGAAATAAAAAAAGAT

Optimized 241 CTGCCACAGTTCATCCTCGAAGAGACCCAGCGCGATGTCCGCTCCATCTTCGAAATCCAC

Original 241 CTTCCTCAATTCATTTTAGAAGAAACACAAAGAGATGTACGTTCTATTTTTGAAATTCAT

Optimized 301 AAGATCTCCAAGCTGTACGGCAAGCTCTGCCGCGACAAGCGCATCCTGAAGATCGCCCAG

Original 301 AAAATTAGTAAACTATATGGCAAACTTTGTAGAGATAAGAGAATTTTAAAAATAGCACAA

Optimized 361 CAGCTGCTCGGCTCCAAGGTGTACATCCACCAGTCCCGCGTCAACCTCAAGCCAGGCTTC

Original 361 CAACTTTTGGGAAGTAAGGTCTACATTCATCAATCCCGTGTAAACCTTAAACCTGGATTT

Optimized 421 GATGGCAAGGAATTCTACTGGCACTCCGATTTCGAAACCTGGCACTCCGAGGACGGCATG

Original 421 GATGGTAAAGAATTCTATTGGCATTCTGATTTTGAAACATGGCACTCTGAGGATGGTATG

Optimized 481 CCAAACATGCGCGCTGTGTCCTGCTCCATCTCCCTGACCAAGAACTACGAGTTCAACGGC

Original 481 CCAAACATGCGTGCTGTTTCATGTTCAATTAGTTTGACTAAAAATTATGAATTTAATGGA

Optimized 541 CCACTCATGGTGATCCCAGGCTCCCACAAGGAATTCGTCTCCTGCTCCGGCAAGACCCCA

Original 541 CCCTTGATGGTAATCCCTGGTTCACATAAGGAATTTGTATCTTGCAGTGGGAAAACACCT

Optimized 601 GACGAGCACTACAAGCAGTCCCTGAAGCGCCAGGAAATCGGCACCCCACAGAAGGAATTC

Original 601 GATGAACACTATAAGCAATCTCTAAAAAGACAGGAAATAGGCACTCCTCAAAAAGAATTT

Optimized 661 CTCGAGCAGATGGTGGAGAAGTCCAAGATCGTCTCCGCAAAGGGCGATGCCGGCTCCGTG

Original 661 CTAGAACAAATGGTTGAAAAAAGTAAGATTGTATCTGCAAAAGGAGATGCAGGTTCAGTA

Optimized 721 ATCTTCTTCGACTGCAACATCATGCACGGCTCCAACGGCAACATCACCCCATACCCACGC

Original 721 ATCTTTTTTGATTGTAATATTATGCATGGTTCAAACGGCAATATCACGCCTTATCCTAGA

Optimized 781 TCCAACGCTTTCTTCGTGTACAACTCCATCCACAACACCCTGGTCGATCCATTCTGCGGC

Original 781 AGTAATGCCTTTTTTGTTTATAATAGCATCCATAATACCTTGGTTGATCCATTTTGTGGT

Optimized 841 CTCAACCCACGCCCAACCTACATCGCAGAACGCGAGTTCCTGCCACTCGAACCAGTCGAT

Original 841 TTAAATCCCAGACCTACCTATATTGCAGAAAGAGAATTTTTGCCTCTTGAGCCTGTTGAT

Optimized 901 AACTTCCTGGACTCCAAGTAA

Original 901 AATTTTCTTGATTCAAAATAG

***Sphyngopyxis alaskensis* DSM 13593 *ectD***

Optimized 1 ATGCAGGATCTGTACCCATCCCGCCAGCGCGCTGATGCAGAAATGCGTCCACGTCTCGAC

Original 1 ATGCAAGACCTCTACCCCTCGCGCCAGCGCGCGGACGCCGAAATGCGGCCGCGGCTGGAC

Optimized 61 CCAGTGGTCCACTCCGAGTGGACCAACGATGCTCCAATCTCCGCACGTCAGGCAGCAGCT

Original 61 CCCGTCGTCCATAGCGAATGGACAAACGATGCGCCGATCAGCGCGCGGCAGGCCGCGGCG

Optimized 121 TTCGATCGTGATGGTTACATCGTGCTGGAAGACATCTTCTCCGCCGACGAGGTCGCTTTC

Original 121 TTCGACCGCGACGGCTATATCGTGCTCGAGGATATTTTCTCGGCCGACGAAGTCGCCTTC

Optimized 181 CTCCAGAAGGCAGCCGGCAACCTGCTCGCTGATCCAGCTGCACTGGATGCAGACACCATC

Original 181 CTGCAAAAGGCCGCGGGCAATCTGCTCGCCGATCCGGCGGCGCTCGACGCCGACACGATC

Optimized 241 GTGACCGAACCACAGTCCAACGAGATCCGCTCCATCTTCGAAATCCACGCACAGTCCCCA

Original 241 GTCACCGAGCCGCAAAGCAACGAGATCCGCTCGATCTTCGAGATTCACGCGCAAAGCCCG

Optimized 301 GTCATGGCACGTCTGGCTGCTGATGCTCGCCTGGCTGATGTGGCTCGCTTCCTGCTCGGC

Original 301 GTGATGGCGCGCCTTGCCGCCGATGCGCGGCTCGCCGATGTCGCGCGCTTCCTGCTCGGC

Optimized 361 GATGAAGTCTACATCCACCAGTCCCGCCTGAACTACAAGCCAGGCTTCAAGGGCCGCGAG

Original 361 GACGAGGTTTATATCCACCAGTCGCGGCTGAACTATAAACCCGGTTTCAAGGGCAGGGAG

Optimized 421 TTCTACTGGCACTCCGATTTCGAAACCTGGCACGTGGAAGATGGTATGCCACGTATGCGC

Original 421 TTCTACTGGCACAGCGATTTCGAAACCTGGCATGTCGAGGACGGGATGCCGCGGATGCGC

Optimized 481 GCACTCTCCATGTCCGTCCTGCTCGCTGAAAACACCCCACACAACGGCCCACTGATGGTG

Original 481 GCGCTGTCGATGTCGGTGCTGCTCGCCGAAAACACCCCGCACAACGGCCCGCTGATGGTG

Optimized 541 ATCCCAGGCTCCCACCGTACCTACCTCACCTGCGTGGGCGAGACCCCAGATGACCACTAC

Original 541 ATTCCGGGCTCGCACCGCACCTATCTCACCTGCGTCGGCGAAACCCCCGACGACCATTAT

Optimized 601 CTGTCCTCCCTCAAGAAGCAGGAATACGGCGTGCCAGATGAAGAATCCCTGGCAGAGCTG

Original 601 TTAAGCTCGCTCAAGAAACAGGAATATGGCGTGCCCGACGAGGAAAGCCTCGCCGAACTG

Optimized 661 GCACACCGTCACGGTATCGTGGCACCAACCGGCAAGCCAGGCACCGTCATCCTGTTCGAC

Original 661 GCGCACAGGCACGGCATCGTCGCGCCGACGGGCAAGCCGGGAACGGTGATCCTGTTCGAC

Optimized 721 TGCAACCTCATGCACGGCTCCAACGGCAACATCACCCCATTCCCACGCGCCAACGCTTTC

Original 721 TGCAATTTGATGCACGGGTCGAACGGCAACATCACGCCCTTTCCGCGCGCCAACGCCTTC

Optimized 781 CTGGTGTACAACGCAGTCTCCAACCGCCTCGAAAAGCCATTCGGCGTGGAGAAGCCACGC

Original 781 CTCGTCTATAATGCCGTGAGCAACCGGCTCGAAAAGCCCTTCGGCGTCGAAAAGCCGCGC

Optimized 841 CCATGGTTCTTGGCACGCCGCGGCGAACCAGCAGCATTGCGTGTGGAGCGCGGTCCACTC

Original 841 CCCTGGTTCCTCGCCCGTCGCGGCGAGCCCGCGGCGCTCCGGGTTGAGCGCGGGCCGCTC

Optimized 901 GTGGAGACCGTCCCAGCATAA

Original 901 GTCGAAACGGTGCCGGCATGA

***Paenibacillus lautus* E7593-69 *ectD***

Optimized 1 ATGTCCAAGAACCACGCATCCGCCCTGCAGGAAAAGGAGATGGATGTGTACCCATCCCGC

Original 1 ATGAGTAAGAATCATGCGTCGGCCCTTCAGGAAAAAGAAATGGACGTCTATCCTTCAAGA

Optimized 61 GTCCACGCTGAACCACGCATCCTGAAGCGCCAGGACCCAGTGGTCCACTCCGAGTGGACC

Original 61 GTGCATGCGGAACCCCGGATCTTAAAAAGGCAGGATCCGGTCGTACATTCGGAGTGGACG

Optimized 121 CCAGATGCACCACTCACCCAGGAACAGTCCGACTTCTACGAGCGCAACGGCTACCTGTTC

Original 121 CCGGATGCGCCCCTAACGCAGGAGCAATCCGACTTCTACGAGCGCAACGGATATTTATTT

Optimized 181 CTCGAAGGCTTCTTCGATCGCGAAGAGCTGTCCCGCTACCAGGAAGAGGCCCGCCGCCTC

Original 181 CTGGAAGGCTTCTTTGATCGGGAGGAGCTTTCGCGATATCAGGAGGAGGCCCGCAGGCTG

Optimized 241 CAGATCACCGCTCGTGAATCCGAGAAGGATGAAGTGATCCGTGAGCCAGGCGGCGACGAA

Original 241 CAGATAACGGCGCGGGAATCGGAGAAAGATGAAGTGATCCGGGAGCCCGGCGGAGACGAA

Optimized 301 GTGCGCTCCGTCTTCGCAGTGCACGAATCCGACGAGGTCTTCAAGAAGCTGTCCCAGCAC

Original 301 GTGCGGTCGGTATTCGCGGTACATGAGAGCGACGAAGTGTTCAAGAAGCTGTCCCAGCAT

Optimized 361 CCACGCCTGCTCGCCATCATGGAATACCTGCTCGGCTCCGAGACCTACATCCACCAGTCC

Original 361 CCTAGACTCCTGGCGATCATGGAATACCTCTTGGGAAGCGAGACGTATATCCATCAATCC

Optimized 421 CGCATCAACTACAAGCCAGGCTTCACCGGCAAGGAATTCTACTGGCACTCCGATTTCGAA

Original 421 CGAATTAATTACAAGCCGGGCTTTACGGGCAAGGAGTTCTACTGGCACTCCGATTTCGAA

Optimized 481 ACCTGGCACGTGGAGGATGGTATGCCACGTATGCGTGCTCTGTCCTGCTCCATCGCACTC

Original 481 ACGTGGCATGTCGAGGACGGCATGCCGCGCATGAGAGCGCTAAGCTGTTCCATTGCCCTG

Optimized 541 GAGGATAACTACCCATACAACGGCCCACTCATGGTGGTCCCAGGCTCCCACAAGGAATTC

Original 541 GAGGACAACTATCCATATAACGGGCCGCTTATGGTGGTGCCCGGATCGCATAAGGAATTC

Optimized 601 GTGGCCTGCATCGGCCAGACCCCAGAGGATCACTTCAAGGACTCCCTGCGCAAGCAGGAA

Original 601 GTCGCCTGCATCGGCCAAACCCCGGAGGATCATTTCAAGGATTCCCTGCGCAAGCAGGAG

Optimized 661 TACGGCGTGCCAGATCACGACTCCCTCACCCGCATGGTCAAGGAAGGCGGCATCGATACC

Original 661 TACGGCGTTCCGGATCATGACAGCCTGACGCGGATGGTGAAGGAGGGCGGAATTGACACG

Optimized 721 CCAGTGGGCAACGCTGGCTCCATCGTCATCTTCGACTGCAACATCATGCACGGCTCCAAC

Original 721 CCGGTCGGCAACGCGGGCTCGATCGTGATTTTTGACTGCAACATTATGCACGGCTCGAAC

Optimized 781 TCCAACATCACCCCAATGCCACGCTCCAACATCTTCATGGTCTACAACTCCGTGGAAAAC

Original 781 AGCAACATCACACCGATGCCGCGAAGCAATATCTTTATGGTCTATAACAGCGTGGAGAAC

Optimized 841 AAGGTCAAGCAGCCATACTCCGGCCAGAAGCCACGCCCAGAATACATCGCAACCCGCGAG

Original 841 AAGGTCAAGCAGCCGTATTCGGGCCAGAAGCCGAGACCGGAGTATATCGCCACCCGTGAG

Optimized 901 TCCCTGTAA

Original 901 TCTCTATGA

***Chromohalobacter salexigens* DSM 3043 *ectD***

Optimized 1 ATGGCAGTGTTCGTCGGCGCCGATCTGTCCGACTACGTGTTCTCCGGTATCGGCGGCAAC

Original 1 ATGGCAGTGTTTGTCGGCGCCGACCTCTCCGACTACGTTTTCTCGGGGATCGGCGGCAAT

Optimized 61 ACCGTCCCATCCCGCCTGATGGAAGAGTTCGCCATGAAGGAAACCCAGGATCTCTTCCCA

Original 61 ACCGTTCCCAGCAGACTAATGGAGGAGTTTGCAATGAAAGAGACACAAGACCTGTTTCCG

Optimized 121 ACCCGCCTGGAACGCAAGCTCGGCATGTTCGAGCGCATCGATCCAGTGGTGCACTCCGAA

Original 121 ACGCGCCTGGAACGCAAACTGGGAATGTTCGAGCGCATCGATCCGGTCGTACACAGTGAA

Optimized 181 GGTGACCAGCGTAAGGGTCCACTGTCCGAAGCTGAGCTCGATGAATTCGACCGCAAGGGC

Original 181 GGCGATCAGCGCAAGGGGCCGCTCAGCGAAGCCGAGCTCGACGAGTTCGACCGCAAGGGG

Optimized 241 TTCCTGTCCTTCGAGGGCTTCTTCGATGAAGACGAGATGGAAGCATTCCTGCAGGAACTC

Original 241 TTCCTGTCTTTCGAGGGGTTCTTCGACGAGGACGAAATGGAAGCGTTCCTCCAGGAGCTC

Optimized 301 CGCGACTACGAGTCCGATGAAGACCTGAAGCTCTCCGAGGGCACCATCCTCGAACCAGGC

Original 301 CGCGACTACGAGAGCGATGAAGACCTCAAGCTCTCGGAAGGCACCATTCTCGAGCCCGGC

Optimized 361 AAGCAGGAGATCCGCTCCATCTTCGGCATCCACGAGGTGTCCGAACGTTTCTCCCGCCTG

Original 361 AAGCAGGAAATCCGTTCGATCTTCGGCATCCACGAGGTGTCAGAACGTTTCAGTCGTCTG

Optimized 421 ACCCGCGATCCACGCCTGCTCGCTATGGTCCAGCAGATCCTCGGCGGCGACGCATACATC

Original 421 ACGCGCGATCCACGCCTATTGGCCATGGTGCAACAGATCCTCGGTGGCGATGCCTACATT

Optimized 481 CACCAGTCCCGCATCAACTACAAGCCAGGCTTCAAGGGCAAGGGCTTCGATTGGCACTCC

Original 481 CACCAATCGCGGATCAACTACAAGCCGGGCTTCAAGGGCAAGGGCTTCGACTGGCATTCG

Optimized 541 GACTTCGAAACCTGGCACTCCGAGGATGGCATGCCACGCATGCGCTCCGTGTCCTGCTCC

Original 541 GATTTCGAGACCTGGCACAGCGAGGACGGCATGCCGCGCATGCGCTCGGTGAGCTGCTCG

Optimized 601 ATCATCCTGACCGAGAACGGCGAATTCAACGGCCCACTGATGCTCGTCCCAGGCTCCCAC

Original 601 ATCATTCTCACCGAAAACGGCGAGTTCAACGGTCCGCTGATGCTGGTGCCCGGTTCGCAC

Optimized 661 CACTACTTCGTGCCATGCGTCGGCCGCACCCCAGAAGATAACTACAAGGAGTCCCTGAAG

Original 661 CATTATTTCGTGCCCTGCGTGGGGCGTACGCCGGAGGACAACTACAAGGAGTCGCTGAAG

Optimized 721 TCCCAGGACATCGGCGTGCCAGATGACGCATCCCTCCGCGATCTGATGCTCAAGGGCGAC

Original 721 AGTCAAGACATCGGCGTGCCGGACGATGCCAGCCTGCGCGACCTGATGCTCAAGGGCGAT

Optimized 781 ATCGAAGCTCCAAAGGGTCCAGTGGGCTCCCTGGTCATGTTCGAGTGCAACACCCTCCAC

Original 781 ATCGAAGCCCCCAAGGGTCCCGTCGGGTCGCTGGTGATGTTCGAGTGCAACACCCTGCAC

Optimized 841 GGCTCCAACATCAACATGTCCTGCTGGCCACGCTCCAACCTGTTCTTCGTGTACAACTCC

Original 841 GGCTCCAACATCAACATGTCGTGCTGGCCGCGCAGCAACCTGTTCTTCGTCTACAACAGT

Optimized 901 GTCGAAAACACCCTCCACGATCCATACTGCGGTAACCGCCCACGTCCAGAGTTCCTGGCA

Original 901 GTCGAGAACACGCTGCACGACCCGTATTGCGGCAACCGTCCGCGGCCCGAGTTCCTCGCC

Optimized 961 AACCGTAAGGACTGGCGTCCACTCACCCCAGCTGAATAA

Original 961 AACCGCAAGGACTGGCGGCCGCTGACACCGGCCGAGTAA
